# From diet to disease modulation: the multi-targeted effects of medicinal-food homologous plants in hepatic fibrosis

**DOI:** 10.3389/fnut.2026.1752309

**Published:** 2026-07-15

**Authors:** Shiqi Chen, Hongdao Chen, Jiaqi Xie, Xiaoyu Peng, Shuhan Yang, Dehui Yin, Ye Zhu

**Affiliations:** 1 Key Laboratory of Emergency and Trauma of Ministry of Education, Department of Chinese Medicine, The First Affiliated Hospital, Hainan Medical University, Haikou, Hainan, China; 2 College of Chinese Medicine, Hainan Medical University, Haikou, Hainan, China

**Keywords:** bioactive compounds, hepatic fibrosis, mechanism of action, medicinal-food homology, treatment

## Abstract

Hepatic fibrosis is a pivotal pathological stage in chronic liver disease progression for which treatment options remain limited. Medicinal-food homologous (MFH) plants, recognized for their dual safety and bioactivity, offer promising preventive and therapeutic potential. This review systematically analyzes the anti-hepatofibrotic mechanisms of 102 officially approved MFH plants. Based on a literature search using keywords such as “specific MFH plant names” and “hepatic fibrosis or liver fibrosis,” we identified 101 relevant studies. The analysis reveals that these plants exert their effects through multi-target synergy, mitigating oxidative stress and inflammation, inhibiting hepatic stellate cell (HSC) activation, inducing activated HSC apoptosis, and modulating the gut-liver axis. Although bioactive compounds such as hesperetin, puerarin, and curcumin have emerged as prominent candidates for therapeutic intervention in hepatic fibrosis, related studies remain largely confined to the pre-clinical stage. Furthermore, approximately one-third of the evidence is derived from crude extracts with poorly characterized compositions; active compounds lack systematic pharmacokinetic and toxicological evaluations; clinical studies remain exceptionally limited; and experimental doses far exceed typical daily dietary intake levels. These limitations severely compromise their clinical translation and application value. Future research should focus on elucidating the mechanisms of single active compounds, establishing long-term intervention models using physiologically relevant doses, and conducting high-quality randomized controlled trials, thereby facilitating the translation of MFH plants from dietary prevention to clinical therapy.

## 1 Introduction

Hepatic fibrosis is a pathological scarring process that arises from an imbalanced wound-healing response to chronic inflammatory stimuli in various chronic liver diseases, such as chronic hepatitis B/C, non-alcoholic fatty liver disease, alcoholic liver disease, autoimmune liver diseases, and cholestatic disorders (1). It is characterized by excessive deposition of extracellular matrix (ECM) components, primarily driven by the activation of hepatic stellate cell (HSC) (2). According to the Global Burden of Disease Study 2023, liver diseases cause approximately 2 million deaths worldwide each year. The condition typically progresses from hepatic fibrosis to cirrhosis and may eventually lead to death due to liver failure, complications of portal hypertension, or hepatocellular carcinoma (HCC) (3). Cirrhosis has become the 11th leading cause of death globally, yet hepatic fibrosis represents a reversible window of opportunity (4).

Existing therapeutic modalities, including antiviral and anti-inflammatory medications, fail to directly address the core pathogenesis of hepatic fibrosis (5). The advancement of multiple anti-hepatofibrotic therapies into Phase III trials, including simtuzumab, selonsertib, TLR4 antagonists, and cenicriviroc, has been followed by the observation of suboptimal efficacy (6). Although significant progress has been made in elucidating the molecular mechanisms of hepatic fibrosis in recent years, only a limited number of drug candidates have advanced into clinical trials, many of which face issues such as unclear safety profiles (7). Thus, the development of safer, more effective, and well-tolerated long-term interventions against hepatic fibrosis, particularly utilizing natural products or functional food components, has become an urgent priority and a hot topic in international liver research (8).

While the term “medicinal-food homology” is modern, its conceptual origins can be traced back to Huangdi Neijing (9). In China, substances traditionally used as both food and medicine are strictly regulated. Their use and quality must comply with the national catalog of substances traditionally used as both food and Chinese Materia Medica and the standards of the Pharmacopeia of the People's Republic of China. Beyond their nutritional value, medicinal-food homologous plants are gaining interest for their multi-target capabilities, high safety, and low toxicity (10). This concept of “medicinal-food homology” aligns with modern preventive medicine and offers a promising strategy to complement conventional therapies against chronic diseases such as liver disorders (11).

The decision to investigate medicinal-food homologous (MFH) plants for hepatic fibrosis is strategically grounded in the unique pathophysiological and clinical attributes of this condition. Unlike acute or self-limiting diseases, hepatic fibrosis represents a chronic, progressive pathological process, thereby creating a critical, extended window for intervention. The chronic progression of hepatic fibrosis is highly compatible with the long-term, low-dose dietary supplementation paradigm of MFH plants. Their established safety profile thereby positions them as well-suited for sustainable management. Furthermore, the complex pathogenesis of hepatic fibrosis includes oxidative stress, chronic inflammation, HSC activation, and gut-liver axis dysregulation. The inherent multi-component nature of MFH plants positions them uniquely to address this complexity synergistically, unlike single-target pharmaceutical agents.

However, a comprehensive and systematic review focusing on the mechanisms and applications of these plants specifically in hepatic fibrosis is still lacking. Therefore, this review aims to synthesize current evidence from studies published between 2010 and 2025, sourced primarily from PubMed, with a focus on the 102 plants listed in the “Regulations on the Management of the Catalog of plants that are Traditionally Both Food and Chinese Medicinal Herbs” issued by China's National Health Commission in November 2021 (10). The complete list of the 102 MFH plants utilized in this study is provided in (12). We focus on their molecular targets, mechanistic pathways, and potential clinical applications in hepatic fibrosis treatment. The Medicinal Plant Names Services was consulted to standardize the nomenclature of all medicinal-food homologous plants (Table 1). By providing a thorough analysis of pre-clinical and clinical findings, this work seeks to support the development of novel therapeutic strategies for mitigating hepatic fibrosis.

**Table 1:** Summary of Medicinal-food homologous (MFH) plants with potential anti-liver fibrosis effects.

Botanical name (Chinese name)	Family name	Part used	Compound	Model	Dosage	Main effects	Molecular mechanisms	References
Alleviating oxidative stress and/or suppressing inflammation								
*Citrus reticulata* Blanco (Jupi)	Rutaceae	Mature pericarp	Hesperetin	HFD-induced Wistar rats; HepG2 cells	100, 300 mg/kg; 2.5, 5, 10 μm	Anti-oxidative stress/anti-inflammatory response	PI3K/AKT-Nrf2-ARE pathway	(1)
*Citrus reticulata* Blanco (Jupi)	Rutaceae	Mature pericarp	Hesperidin	TAA-induced albino rats	200 mg/kg	Anti-oxidative stress/anti-inflammatory response	TGF-β/α-SMA pathways	(2)
*Citrus reticulata* Blanco (Jupi)	Rutaceae	Mature pericarp	Hesperidin	BDL-induced Wistar rats	100, 200 mg/kg	Anti-oxidative stress/anti-inflammatory response	Not mentioned	(3)
*Citrus reticulata* Blanco (Jupi)	Rutaceae	Mature pericarp	Hesperidin	CCl4-induced Wistar rats	200 mg/kg	Anti-oxidative stress/anti-inflammatory response	Not mentioned	(4)
*Citrus reticulata* Blanco (Jupi)	Rutaceae	Mature pericarp	Hesperidin	CCl4-induced Wistar rats	100, 200 mg/kg	Anti-oxidative stress/anti-inflammatory response	Not mentioned	(5)
*Pueraria montana* var. lobate (Gegen)	Fabaceae	Root	Puerarin	CCl4-induced Wistar rats	200, 400, 800 mg/kg	Inhibition of inflammatory response	TNF-α/NF-κB pathway	(6)
*Pueraria montana* var. Lobata (Gegen)	Fabaceae	Root	Puerarin	CCl4-induced C57BL/6J mice	100, 200 mg/kg	Inhibition of inflammatory response	Not mentioned	(7)
*Pueraria montana* var. Lobata (Gegen)	Fabaceae	Root	Polysaccharide	CCl4-induced KM mice	100, 200, 400 mg/kg	Anti-oxidative stress/anti-inflammatory response	Not mentioned	(8)
*Codonopsis pilosula* (Dangshen)	Campanulaceae	Root	Polysaccharide	CCl4-induced Kun-ming mice; LX-2 cells	50, 100 mg/kg; 50, 100, 200 μg/ml	Anti-oxidative stress/anti-inflammatory response	TLR4/NF-κB and TGF-β1/Smad3 signaling pathways	(9)
*Lycium barbarum* L. (Gouqizi)	Solanaceae	Mature fruit	Polysaccharides	CCl4-induced Wistar rats	400, 800, 1,600 mg/kg	Anti-inflammatory response	TLR/NF-κB axis	(10)
*Polygonatum sibiricum* (Huangjing)	Asparagaceae	Rhizome	Polysaccharide	HFD + 20% ethanol + CCl4-induced SD rats	0.8, 1.6 g/kg	Anti-oxidative stress/anti-inflammatory response	TGF-β/Smad signaling pathway	(11)
*Astragalus mongholicus* (Huangqi)	Leguminosae	Root	Polysaccharide	Alcohol-induced SD rats	200, 400 mg/kg	Inhibition of inflammatory response	TLR4/JNK/NF-κB/MyD88 pathway	(12)
*Astragalus mongholicus* (Huangqi)	Leguminosae	Root	Flavonoids	HSC-T6	1, 5, 10 μm	Inhibition of inflammatory response	NF-κB signal pathway	(13)
*Astragalus mongholicus* (Huangqi)	Leguminosae	Root	Cycloastragenol	CCl4-induced ICR outbred mice	50, 200 mg/kg	Inhibition of inflammatory response	TGF-β1/Akt signaling pathway	(14)
*Dioscorea oppositifolia* L. (Shanyao)	Dioscoreaceae	Rhizome	Trillin	CCl4-induced C57BL/6 mice	50, 100 mg/kg	Inhibition of inflammatory response	NF-κB pathway and TGF-β/Smad pathway	(15)
*Crataegus pinnatifida* (Shanzha)	Rosaceae	Mature fruit	Corosolic acid	HC-induced C57BL/6J mice; HepG2 cells, LX2 cells	10, 20 mg/kg; 5, 10, 20 μm	Inhibition of inflammatory response	TGF-β1/Smad2, NF-κB, and AMPK signaling pathways	(16)
*Crataegus pinnatifida* (Shanzha)	Rosaceae	Mature fruit	Hyperoside	CCl4-induced C57BL/6J mice;	100 and 200 mg/kg	Anti-oxidative stress/anti-inflammatory response	HMGB1-TLR4-NF-κB pathway	(17)
*Angelica dahurica* (Baizhi)	Apiaceae	Root	Bergapten	CCl4-induced Kunming mice; HSC-T6, LX-2 cells	100, 200, 300 mg/kg; 3.125, 6.25, 12.5 μm	Inhibition of inflammatory response	FXR signaling pathway	(18)
*Angelica dahurica* (Baizhi)	Apiaceae	Root	Imperatorin	CCl4-induced SD rats; LX-2 cells	15, 25 mg/kg; 100 μm	Inhibition of inflammatory response	TGF-β signaling pathway	(19)
*Wolfiporia cocos* (Fuling)	Polyporaceae	Sclerotium	Dehydrotrametenolic acid methyl ester	HFD + CCl4-induced C57BL/6J mice; Primary mouse cells	30, 75 mg/kg; 5, 10, 20 μm	Inhibition of inflammatory response	Caspase-1/NLRP3 axis	(20)
*Lonicera japonica* (Jinyinhua)	Caprifoliaceae	Flower bud	Water extract	CCl4-induced C57BL/6 mice;	0.2, 0.4 g/kg	Anti-oxidative stress	Nrf2 pathway	(21)
*Lonicera japonica* (Jinyinhua)	Caprifoliaceae	Flower bud	Water extract	TAA-induced Wistar rats; FL83 B cells	2.5 ml/kg; 5, 10, 20, 50, 100 μg/ml	Anti-oxidative stress	Not mentioned	(22)
*Lonicera japonica* (Jinyinhua)	Caprifoliaceae	Flower bud	Chlorogenic acid	CCl4-induced SD rats	20, 40, 60 mg/kg	Anti-oxidative stress/anti-inflammatory response	PI3K/AKT/mTOR signaling pathway	(23)
*Lonicera japonica* (Jinyinhua)	Caprifoliaceae	Flower bud	Chlorogenic acid	CCl4-induced SD rats, LX2 cells	15, 30, 60 mg/kg; 20?40?80 μg/ml	Anti-oxidative stress/anti-inflammatory response	miR-21-regulated TGF-β1/Smad7 signaling pathway	(24)
Fructus Mori (Sangshen)	Moraceae	Multiple fruit	Aqueous extracts	CCl4-induced ICR mice; HepG2 cells	100, 200 mg/kg; 20 μg/ml	Anti-oxidative stress/anti-inflammatory response	Nrf2/HO-1 pathway	(25)
*Platycodon grandiflorum* (Jiegeng)	Campanulaceae	Root	Aqueous extract	BDL-induced SD rats	10, 50 mg/kg	Anti-oxidative stress/anti-inflammatory response	Not mentioned	(26)
*Platycodon grandiflorum* (Jiegeng)	Campanulaceae	Root	Aqueous extract	DMN-induced SD rats	100 mg/kg	Anti-oxidative stress	Not mentioned	(27)
*Platycodon grandiflorum* (Jiegeng)	Campanulaceae	Root	Platycodin D	BDL-induced ICR mice	1, 2, and 4 mg/kg	Anti-oxidative stress	Not mentioned	(28)
*Portulaca oleracea* L. (Machixian)	Portulacaceae	Aerial part	Extract (POL-1)	CCl4-induced Kun-ming mice; LX-2 cells	50, 200 mg/kg; 30, 50, 100 μg/ml	Anti-oxidative stress/anti-inflammatory response	Bcl-2/Bax and TGF-β1/Smad2 signaling pathway	(29)
Folium Mori (Sangye)	Moraceae	Leaf	Extracts	HFD-induced C57BL/6 mice	133, 666 mg/kg	Anti-Oxidative stress	Not mentioned	(30)
Folium Mori (Sangye)	Moraceae	Leaf	1-Deoxynojirimycin	db/db mice	25, 50, 100 mg/kg	Anti-Oxidative stress	AMPK/SIRT1 signaling pathway	(31)
*Chrysanthemum morifolium* (Juhua)	Asteraceae	Capitulum	Water extract	MCD diet-induced C57BL/6 mice;HepG2 cells	200, 400 mg/kg; 1, 5, 10 μg/ml	Inhibition of inflammatory responses	Not mentioned	(32)
*Rubus chingii* (Fupenzi)	Rosaceae	Fruit	Ellagic acid	Iron dextran-induced C57BL/6 mice; AML12 cells	25, 50 mg/kg; up to 20 μm	anti-oxidative stress and alleviating ferroptosis	TGFβ/Smad signaling pathway	(33)
*Rubus chingii* (Fupenzi)	Rosaceae	Fruit	Ellagic acid	TiO_2_ NPs-induced C57BL/6 mice; L02 cell	25, 50, 100 mg/kg; 45 μm	Anti-oxidative stress/anti-inflammatory response	Nrf2 signaling pathway	(34)
*Gardenia jasminoides* (Zhizi)	Rubiaceae	Mature fruit	Geniposide	CCl4-induced C57BL/6 mice	50 mg/kg	Anti-oxidative stress/anti-inflammatory response	Not mentioned	(35)
*Gardenia jasminoides* (Zhizi)	Rubiaceae	Mature fruit	Geniposide	HFD-induced C57BL/6 mice	50, 100 mg/kg	Anti-inflammatory response	INSR-IRS2-Akt insulin signaling pathway	(36)
*Cornus officinalis* (Shanzhuyu)	Cornaceae	Fruit	Loganin	MCD-induced C57BL/6 mice	5, 30 mg/kg	Anti-inflammatory response	Not mentioned	(37)
*Citrus aurantium* L (Daidaihua)	Rutaceae	Flower bud	Naringin	MCD-induced C57BL/6 mice	100, 200 mg/kg	Anti-oxidative stress/anti-inflammatory response	Not mentioned	(38)
*Citrus aurantium* L (Daidaihua)	Rutaceae	Flower bud	Naringin	HFD-induced C57BL/6 mice	25, 50 mg/kg	Anti-inflammatory response	Not mentioned	(39)
*Siraitia grosvenorii* (Luohanguo)	Cucurbitaceae	Fruit	Extract (main component: mogrosides)	CDAA-HF-T (-) diet-induced C57BL/6J mice	0.2%, 0.6%, and 2% in drinking water	Anti-oxidative stress/anti-inflammatory response	Not mentioned	(40)
*Taraxacum mongolicum* (Pugongying)	Asteraceae	Herb	Taraxasterol	CCl4-induced Kunming mice	2.5, 5, 10 mg/kg	Anti-inflammatory response	Hippo, HIF-1α, TGF-β/Smad, and Wnt pathway	(41)
Inhibition of hepatic stellate cell activation and Induction of their apoptosis								
*Siraitia grosvenorii* (Luohanguo)	Cucurbitaceae	Fruit	Mogroside IVE	CCl4-induced C57BL/6 mice; HSC-T6	25 mg/kg; 0.5, 1, 10 μm	Inhibiting HSC activation	TLR4/HIF-1 α cohort signaling pathway	(42)
*Myristica fragrans* (Roudoukou)	Myristicaceae	Seed kernel	Methoxyeugenol	CCl4-induced BALB/c mice; HepG2 cells	0.25, 1.0 mg/kg; 15, 30, 60, 125, 250 μm	Inhibiting HSC activation	PPAR-γ/NF-κB axis	(43)
*Cistanche deserticola* (Roucongrong)	Orobanchaceae	Succulent stems	Phenylethanol Glycosides	HSC-T6	100, 50 μg/ml	Inhibiting HSC activation	TGF-β1/smad pathway	(44)
*Cistanche deserticola* (Roucongrong)	Orobanchaceae	Succulent stems	Echinacoside	TAA-induced SD rats	15, 30, 45, 60 mg/kg	Not mentioned	Not mentioned	(45)
*Ziziphus jujube* (Dazao)	Rhamnaceae	Mature fruit	Spinosin	CCl4-induced C57BL/6 mice; AML12,LX-2 cells	20, 40 mg/kg; 10, 20 μm	Inhibiting HSC activation	Nur77/ASK1/p38 MAPK pathway	(46)
*Lonicera japonica* (Jinyinhua)	Caprifoliaceae	Flower bud	Sweroside	CCl4-induced C57BL/6 mice, FXR knockout mice; LX-2 and L02 Cells	125 mg/kg; 50 μm	Inhibiting HSC activation	FXR-miR-29a signaling pathway	(47)
*Lonicera japonica* (Jinyinhua)	Caprifoliaceae	Flower bud	Chlorogenic acid	MCD diet-induced C57BL/6 mice; LX2 cells	30, 60 mg/kg; 1, 5, 10 μm	Inhibiting HSC activation	PGC1α/NRF1 pathway	(48)
*Platycodon grandiflorum* (Jiegeng)	Campanulaceae	Root	Platycodin D	CCl4-induced C57BL/6 mice; LX2 cells	4, 8 mg/kg; 20, 40, 80 μm	Inhibiting HSC activation	JNK/c-Jun signaling pathway	(49)
Fructus Mori (Sangshen)	Moraceae	Multiple fruit	Mulberry marc anthocyanins	CCl4-induced SD rats	200, 400, 800 mg/kg	Inhibiting HSC activation	Not mentioned	(50)
*Hovenia dulcis* (Zhijuzi)	Rhamnaceae	Mature fruit	Dihydromyricetin	LX-2 cells	10, 30, 50 μm	Inhibiting HSC activation	mTOR/MAPK pathway	(51)
*Hovenia dulcis* (Zhijuzi)	Rhamnaceae	Mature fruit	Dihydromyricetin	CCl4-induced C57BL/6 mice; LX2 cells	100 mg/kg; 0, 10, 30, 50 μm	Inhibiting HSC activation	AhR-NF-κB/STAT3-IFN-γ signaling pathway	(52)
*Hovenia dulcis* (Zhijuzi)	Rhamnaceae	Mature fruit	Total flavonoids	CCl4-induced Kunming mice; HSC-T6 cells	150, 300, 450 mg/kg; 5, 10, 20, and 30 μg/ml	Apoptosis of activated HSC	PI3K/AKT Signaling Pathway	(53)
*Gardenia jasminoides* (Zhizi)	Rubiaceae	Mature fruit	Novel polysaccharide	CCl4-induced C57 mice; LX-2 cells	50, 100 mg/kg; 0.1, 0.2, 0.4, 0.8, 1.0 mg/ml	Inhibiting HSC activation	TLR4/NF-κB signaling	(54)
*Gardenia jasminoides* (Zhizi)	Rubiaceae	Mature fruit	Extracts	BDL-induced SD rats; LX-2 cells	25, 50, 100 mg/kg; 20, 40, 80 μmol/L	Inhibiting HSC activation	TGF-β1/smad2 pathway	(55)
*Gardenia jasminoides* (Zhizi)	Rubiaceae	Mature fruit	Geniposide	CCl4-induced Kunming mice; HSC-T6 cells	50, 100, 150 mg/kg; 25, 50, 100 μm	Inhibiting HSC activation	Sonic hedgehog (Shh) signaling pathway	(56)
*Perilla frutescens* (Zisu)	Lamiaceae	Leaf	Luteolin-7-diglucuronide	Diet-combined CCl4-induced C57BL/6 mice; pHSCs, LX-2 cells	50, 100 mg/kg; 5, 20, 50 μm	Inhibiting HSC activation	PTP1B-AMPK signaling pathway	(57)
*Pueraria montana* var. Lobata (Gegen)	Fabaceae	Root	Puerarin	DMN-induced SD rats	100, 200 mg/kg	Inhibiting HSC activation	TGF-β1/Smad signaling pathway	(58)
*Hippophae rhamnoides* L. (Shaji)	Elaeagnaceae	Mature fruit	Extracts	BDL-induced SD rats; Rat HSCs	20, 40 mg/kg; 20, 40, 80, 160, 320 μm	Inhibiting HSC activation	Not mentioned	(59)
Semen Persicae (Taoren)	Rosaceae	Mature fruit	Extracts	HSC-T6	10, 50, 100 μg/ml	Inhibiting HSC activation	Not mentioned	(60)
Semen Persicae (Taoren)	Rosaceae	Mature fruit	Amygdalin	CCl4-induced C57BL/6 mice; LX-2 cells	100 mg/kg; 25, 50, 100 μm	Inhibiting HSC activation	mTOR/PDCD4/JNK pathway	(61)
Semen Persicae (Taoren)	Rosaceae	Mature fruit	Amygdalin	CCl4-induced SD rats; LX-2 cells	3 mg/kg; 1.25, 2.5, 5.0 mg/ml	Inhibiting HSC activation	TGF-β1/Smad2/3 and NF-κB p65 signaling pathways	(62)
*Cornus officinalis* (Shanzhuyu)	Cornaceae	Fruit	Morroniside	CCl4 and HFD-induced C57BL/6 mice; HSC-T6 cell	5, 10, 20 μm	Inhibiting HSC activation	GATA/Lipa signaling pathway	(63)
HPWS forms-*Cornus officinalis* (Shanzhuyu)	Cornaceae	Fruit	Extracts	HSC-T6, HK-2 cells	10 mg/ml	Inhibiting HSC activation/Apoptosis of activated HSC	SIRT3/AMPK axis	(64)
*Glycyrrhiza inflate* (Gancao)	Fabaceae	Roots and Rhizomes	18 beta-glycyrrhetinic acid	BDL-induced C57BL/6 mice; LX-2 cells	20, 50 mg/kg; 20, 40, 80 μm	Apoptosis of activated HSC	Not mentioned	(65)
*Glycyrrhiza inflate* (Gancao)	Fabaceae	Roots and Rhizomes	Glycyrrhetinic acid	LX2 cells	10, 15 μm	Inhibiting HSC activation	TGF-β/Smad Signaling Pathway	(66)
*Citrus reticulata* Blanco (Jupi)	Rutaceae	Mature pericarp	Hesperetin	BDL-induced C57 mice	200 mg/kg	Apoptosis of activated HSC	TGF-β1/Smad Pathway	(67)
*Crataegus pinnatifida* (Shanzha)	Rosaceae	Mature fruit	Hyperoside	LX2 cells	0.5, 1.0, 2.0 μm	Apoptosis of activated HSC	NF-κB Pathway	(68)
*Ganoderma lucidum* (Lingzhi)	Polyporaceae	Fruiting body	*Ganoderma lucidum* polysaccharide	CCl4-induced C57BL/6 mice; HSC-T6 cells	150, 300 mg/kg; 1.25, 2.5, 5 mg/ml	Inhibiting HSC activation/Apoptosis of activated HSC	TLR4/NF-κB/MyD88 and TGF-β/Smad signaling pathways	(69)
*Dendrobium officinale* (Shihu)	Orchidaceae	Stem	*Dendrobium officinale* Polysaccharide	CCl4-induced SD rats; Caco-2 cells	200, 400, 800 mg/kg; 0, 50, 100, and 200 μg/ml	Apoptosis of activated HSC	LPS-TLR4-NF-κB Signaling Pathway	(70)
*Dendrobium officinale* (Shihu)	Orchidaceae	Stem	*Dendrobium officinale* Polysaccharide	CCl4-induced SD rats; HSC-T6 cells	200 mg/kg; 100, 200, 400 μg/ml	Inhibiting HSC activation	SMO/Gli 1 pathway	(71)
*Laminaria japonica* Aresch. (Kunbu)	Laminariaceae	Thallus	Fucoidan	CCl4-induced C57BL/6J mice	100, 200 mg/kg	Inhibiting HSC activation	TLR4/NF-κB Pathway	(72)
*Laminaria japonica* Aresch. (Kunbu)	Laminariaceae	Thallus	Fucoidan oligosaccharides	MOD-induced C57BL/6J mice	100, 200 mg/kg	Inhibiting HSC activation	JAK/STAT3/FUT8 axis	(73)
Modulation of the gut microbiota								
*Cistanche deserticola* (Roucongrong)	Orobanchaceae	Succulent stems	Phenylethanol Glycosides	BSA-Induced SD rats	100 mg/ml	Modulating gut Microbiota-Liver Axis	LPS-TLR4/MyD88/NF-κB pathway	(74)
*Phyllanthus emblica* L. (Yuganzi)	Euphorbiaceae	Mature fruit	Gallic acid, corilagin, and ellagic acid	CDAHFD-induced C57BL/6J mice	0.9, 1.8, 3.6 g of crude drug/kg	Modulating gut Microbiota	Not mentioned	(75)
*Lycium barbarum* L. (Gouqizi)	Solanaceae	Mature fruit	Oligosaccharides	CCl4-induced C57BL/6J mice	200 mg/kg	Modulating gut Microbiota	Not mentioned	(76)
*Lycium barbarum* L. (Gouqizi)	Solanaceae	Mature fruit	Polysaccharides (LRMP1)	CCl4 and MCD-induced C57BL/6J mice	100, 200 mg/kg	Modulating gut Microbiota	Not mentioned	(77)
*Lycium barbarum* L. (Gouqizi)	Solanaceae	Mature fruit	Peptidoglycan	CCl4-induced C57BL/6J mice; LX2 cells	50, 100, 200 mg/kg; 0.25, 0.5, 1.0 mg/ml	Modulating gut Microbiota	TGF-β/Smads pathway	(78)
*Pueraria montana* var. Lobata (Gegen)	Fabaceae	Root	Pueraria lobata polysaccharides	CCl4-induced Kunming mice	200, 400 mg/kg	Modulating gut Microbiota	Nrf2/HO-1/GPX4 axis	(79)
*Cichorium intybus* L. (Juju)	Asteraceae	Aerial parts/Root	Lactucin	CCl4-induced SD rats; HSC-T6 and RAW 264.7 cells	2.5, 10.5 mg/kg; 6.25, 12.5, 25 μg/ml	Modulating gut Microbiota	TLR4-MyD88-MAPK/NF-κB axis	(80)
*Cichorium intybus* L. (Juju)	Asteraceae	Aerial parts/Root	Lactucin	HSC-T6 cells	5, 10, 20 μm	Modulating gut Microbiota	TGF-β1/STAT3 signaling pathway	(81)
*Ganoderma lucidum* (Lingzhi)	Polyporaceae	Fruiting body	Total triterpenoids	CCl4-induced SD rats; LX-2 cells	120, 240, 480 mg/Kg; 2, 5, 10, 25, 50 μg/ml	Modulating metabolites and gut microbiota g_Ruminococcus	NF-κB and TGF-β1/Smads pathways	(82)
*Rubus chingii* (Fupenzi)	Rosaceae	Fruit	*Rubus chingii* Hu. unripe fruits extract	CCl4-induced C57BL/6 mice	450, 900 mg/kg	Modulating gut Microbiota	TGF-β/Smads pathway	(83)
*Crataegus pinnatifida* (Shanzha)	Rosaceae	Mature fruit	Hyperoside	CCl4-induced C57BL/6 mice; LX2 cells	20, 40, 80 mg/kg; 10, 20, 40 μm	Modulating gut Microbiota	Flot2/TLR4/NLRP3 axis	(84)
*Crataegus pinnatifida* (Shanzha)	Rosaceae	Mature fruit	Ursolic acid	MCD and CCl4-induced C57BL/6 mice	40 mg/kg	Modulating gut Microbiota	NOX2/NLRP3 pathway	(85)
*Hovenia dulcis* (Zhijuzi)	Rhamnaceae	Mature fruit	Dihydromyricetin	CCl4-induced C57BL/6 mice	100 mg/kg	Modulating gut Microbiota	Not mentioned	(86)
*Laminaria japonica* Aresch. (Kunbu)	Laminariaceae	Thallus	Fucoidan	Alcohol-induced BALB/c mice	300 mg/kg	Modulating gut Microbiota	NF-κB/MAPK and Nrf2 signaling pathways	(87)
Spice								
*Piper longum* L. (Biba)	Piperaceae	Fruiting spike	Piperlongumine	BDL-induced Swiss albino mice	1.25, 2.5 mg/Kg	Anti-oxidative stress/anti-inflammatory response/inhibition of HSC activation	TGF-β1/Smad and EMT pathways	(88)
*Crocus sativus* L. (Zanghonghua)	Iridaceae	Stigma	Crocin	CCl4-induced SD rats	20, 40, 80 mg/kg	Inhibition of inflammatory response	Not mentioned	(89)
*Crocus sativus* L. (Zanghonghua)	Iridaceae	Stigma	Extracts	CCl4-induced KM mice	50, 100 mg/kg	Inhibition of inflammatory response	Akt/HIF-1α/VEGF Signaling Pathway	(90)
*Kaempferia galanga* L. (Shannai)	Zingiberaceae	Rhizome	Kaempferol	CCl4-induced SD rats; HSC-T6	12.5, 25, 50 mg/kg; 5, 10, 20 μm	Inhibiting HSC activation	ASIC1a-eIF2α-ATF-4 signaling pathway	(91)
*Angelica sinensis* (Danggui)	Apiaceae	Root	Polysaccharide	CCl4-induced C57BL/6J mice; HSCs were isolated from C57BL/6J mice	200 mg/kg; 50, 100, 200 μg/ml	Inhibiting HSC activation	IL-22/STAT3 pathway	(92)
*Angelica sinensis* (Danggui)	Apiaceae	Root	Levistilide A	CCl4-induced C57BL/6J mice; RAW264.7 cells	4.5, 9 mg/kg; 6.25, 50, 100 μm	Inhibition of inflammatory response	NF-κB/iNOS/NO pathway	(93)
*Angelica sinensis* (Danggui)	Apiaceae	Root	Levistilide A	CCl4-induced Wistar rats; LX-2 cell	3, 6 mg/kg; 12.5, 25, 50 μm	Inhibition of angiogenesis	VEGF Signaling Pathway	(94)
*Pogostemon cablin* (Huoxiang)	Lamiaceae	Aerial parts	Pogostone	HFD-induced C57BL/6 J mice; primary hepatocytes	5, 10, 20 mg/kg; 50, 100, 200 μg/ml	Inhibition of inflammatory response	NLRP3 signal pathway	(95)
*Curcuma longa* L. (Jianghuang)	Zingiberaceae	Rhizome	Six potential active substances	CCl4-induced SD rats; LX-2 cells	0.95, 2.85 ml/kg; 25, 50, 100 μm	Apoptosis of activated HSC	PI3K/Akt/mTOR pathway	(96)
*Curcuma longa* L. (Jianghuang)	Zingiberaceae	Rhizome	Curcumol	CCl4-induced ICR mice; LX-2 cells	30 mg/kg; 20, 30, 45 μm	Apoptosis of activated HSC	Sirt1/Notch pathway	(97)
Combined application								
*Perilla frutescens* (Zisu)	Lamiaceae	Leaf	Luteolin and Silibinin	TAA-induced Wistar rats	50 mg/kg + 100 mg/kg	Anti-oxidative stress/anti-inflammatory	Not mentioned	(98)
*Curcuma longa* L. (Jianghuang)	Zingiberaceae	Rhizome	Tetrahydrocurcumin + EW-7,197	MCD-induced C57BL/6J mice; AML-12, LX-2 cell	100 mg/kg + 20 mg/kg; 1 μm + 0.5 μm	Anti-oxidative stress/anti-inflammatory	TGF-β/Smad2/3 pathway	(99)
*Syzygium aromaticum* (Dingxiang)	Myrtaceae	Flower bud	Ethanolic extract + Silymarin	CCl4-induced Wistar rats; HepG-2 cell	40 mg/kg + 50 mg/kg; 6.25, 12.5, 25, 50, 100, and 200 μg/ml	Anti-oxidative stress/anti-inflammatory	TLR4/MyD88/NF-κB pathway	(100)
*Ziziphus jujube* (Dazao)	Rhamnaceae	Mature fruit	*Ziziphus jujuba* seed powder + oil	CCl4-induced SD rats	5 g/kg + 5 ml/kg	Anti-oxidative stress/anti-inflammatory	TGF-β/Smad pathway	(101)

## 2 Pathological mechanism

The pathogenesis of hepatic fibrosis is initiated by parenchymal liver cell injury resulting from various damaging agents, such as alcohol, viruses, or metabolic stress (13). Apoptosis, necrosis, or pyroptosis of hepatocytes or cholangiocytes leads to the release of damage-associated molecular patterns (DAMPs), including HMGB1 and ATP, alongside an excessive accumulation of reactive oxygen species (ROS) triggered by mitochondrial dysfunction (14). These signals further activate pattern recognition receptors (including TLRs and the NLRP3 inflammasome) on resident hepatic immune cells, such as Kupffer cells and liver sinusoidal endothelial cells, triggering innate immune responses and promoting the release of pro-inflammatory cytokines (15). Consequently, neutrophils, monocytes, and lymphocytes are recruited and activated, establishing a chronic inflammatory microenvironment (16).

Furthermore, gut dysbiosis and impaired intestinal barrier function increase intestinal permeability, allowing gut-derived pathogen-associated molecular patterns (PAMPs), such as lipopolysaccharide (LPS), to enter the liver via the portal vein (17, 18). LPS interacts with Toll-like receptor 4 (TLR4), activating transcription factors including NF-κB and IRF3 through MyD88-dependent signaling (19), thereby exacerbating the cytokine storm and providing persistent stimulation for HSC activation (20).

HSC activation is a central event in hepatic fibrosis. Driven by inflammatory cytokines, ROS, LPS, and paracrine signals such as platelet-derived growth factor (PDGF) and transforming growth factor-β1 (TGF-β1), quiescent vitamin A-storing HSC undergo activation (21). This process involves two phases, an “initiation” phase that sensitizes HSC to growth factors and cytokines, and a “perpetuation” phase maintained by autocrine and paracrine mechanisms, sustaining their proliferative, fibrogenic, and contractile phenotype (22). During the initiation phase, activated HSCs first enter an inflammatory and migratory state without significant fibrogenesis (23). Subsequently, in the perpetuation phase, PDGF strongly promotes HSC proliferation via the MAPK and PI3K-Akt pathways (24), while TGF-β1 induces massive deposition of ECM components, such as type I and III collagen, through the Smad2/3 pathway (25).

As fibrosis progresses into the mid-to-late stage, enhanced autophagy provides energy and substrates required for HSC activation (26). Senescent HSC, via the senescence-associated secretory phenotype (SASP), secrete numerous pro-inflammatory and pro-fibrotic mediators that maintain the fibrotic microenvironment in a paracrine manner (27). Excessive ECM deposition coupled with inadequate degradation results in matrix accumulation and increased tissue stiffness (28). An imbalance between matrix metalloproteinases (MMPs) and their inhibitors (TIMPs), particularly upregulation of TIMP1/2, restricts ECM breakdown (29). Increased matrix stiffness activates mechanotransduction pathways via integrin-focal adhesion signaling, promoting nuclear translocation of YAP/TAZ (30). Upon binding to TEAD, YAP/TAZ upregulate pro-fibrotic and proliferative genes (31, 32), leading to further ECM deposition, increased matrix stiffness, and consequently enhanced YAP/TAZ activity (33), thereby establishing a positive feedback loop of ECM deposition to increased stiffness to sustained HSC activation.

**Figure 1:**
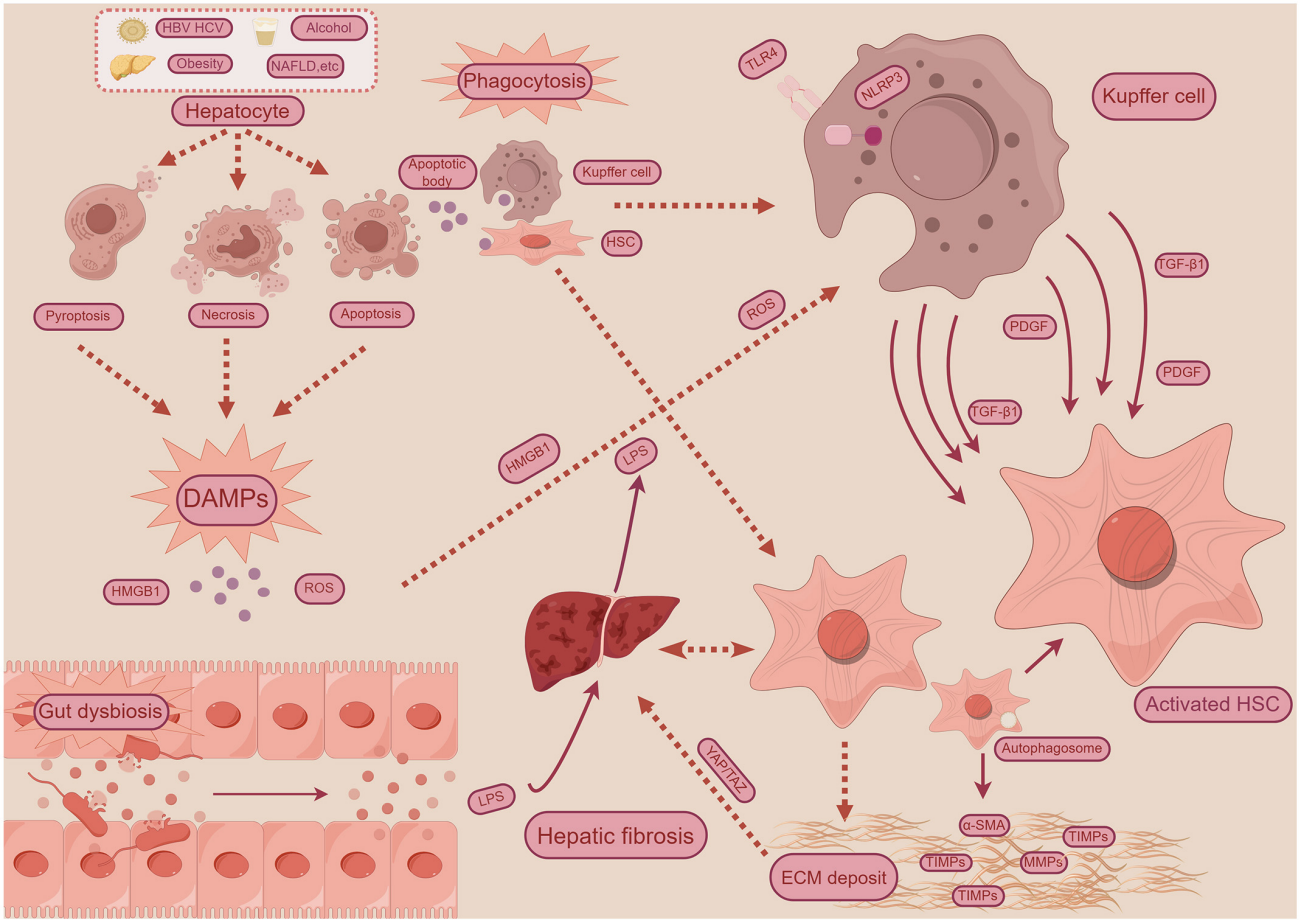
The key pathological mechanisms of hepatic fibrosis. NAFLD, non-alcoholic fatty liver disease; DAMPs, damage-associated molecular patterns; LPS, lipopolysaccharide; TGF-β1, transforming growth factor-β1; PDGF, platelet-derived growth factor; HSC, hepatic stellate cell; α-SMA, α-smooth muscle actin; ECM, extracellular matrix.

In summary, hepatic fibrosis is characterized by the involvement of multiple mechanisms and a variety of cell types (As summarized in Figure 1). This ultimately leads to abnormal ECM accumulation and architectural disruption of the liver. A deeper understanding of these processes provides a theoretical foundation for developing targeted therapies against specific stages of fibrosis.

## 3 Amelioration of oxidative stress and suppression of inflammation

**Figure 2:**
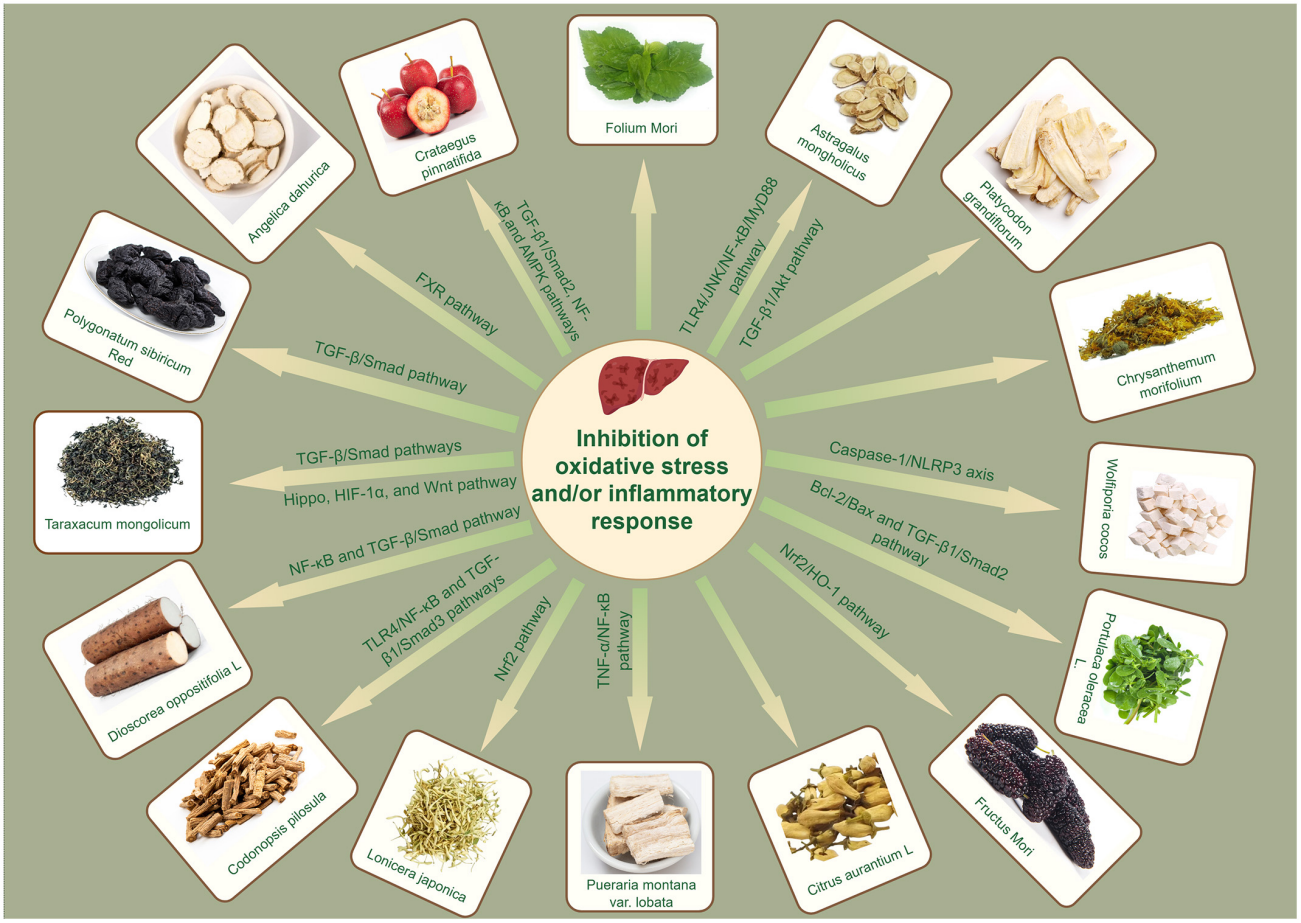
Medicinal-food homologous (MFH) plants ameliorate hepatic fibrosis by targeting oxidative stress and inflammation.

Hepatic fibrosis is driven by a self-perpetuating cycle of oxidative stress and chronic inflammation, each exacerbating the other. Breaking this cycle is therefore a paramount therapeutic objective. Many such MFH plants provide a multi-target intervention strategy for achieving this goal by enhancing the liver's antioxidant capacity and/or suppressing excessive inflammatory responses. This section describes how these medicinal-food homologous plants modulate the above-mentioned mechanism to alleviate hepatic fibrosis (Figure 2).

*Citrus reticulata* Blanco has been relatively well studied; for example, hesperetin ameliorates hepatic oxidative stress and inflammation via the PI3K/AKT-Nrf2-ARE pathway in NAFLD-associated hepatic fibrosis (34). Another bioactive compound, hesperidin alleviates hepatic fibrosis by means of anti-oxidative and anti-inflammatory actions, targeting the TGF-β/α-SMA pathways (35). The anti-hepatofibrotic effects of hesperidin have been consistently demonstrated in multiple rat models of hepatic fibrosis. For example, studies by (36–38) collectively provide evidence for its efficacy across diverse etiologies, spanning chemically-induced models (using dimethylnitrosamine and carbon tetrachloride) and a cholestatic model induced by bile duct ligation.

Another bioactive compound that has been relatively well studied is puerarin from *Pueraria montana* var. lobata. Puerarin may prevent hepatic fibrosis via inhibition of PARP-1 and the subsequent suppression of NF-κB activation, ROS production, and mitochondrial dysfunction (39). Puerarin also alleviates hepatic fibrosis through the suppression of inflammation mediated by the TNF-α/NF-κB signaling pathway (40). In a rat model of comorbid cardiovascular disease and hepatic fibrosis, orally administered puerarin exhibited altered pharmacokinetics, characterized by a significant decrease in systemic exposure compared with the control group, which indicated a substantial reduction in its oral bioavailability. This was correlated with increased expression of the drug transporter P-gp and metabolizing enzymes Ugt1a1/7 in the liver and intestine (41).

Beyond the above extensively studied components, ellagic acid from Rubus chingii (raspberry) also demonstrates multi-dimensional, multi-mechanism anti-hepatofibrotic potential. Studies have shown that ellagic acid alleviates iron overload-induced liver injury and fibrosis by modulating the TGFβ/Smad signaling pathway to inhibit ferroptosis and oxidative stress (42). Another study confirmed that ellagic acid alleviates titanium dioxide nanoparticle-induced hepatic oxidative stress, inflammation, and fibrosis through an Nrf2-dependent pathway (43). More uniquely, ellagic acid alleviates liver fibrosis by inducing FPN-dependent ferroptosis in activated hepatic stellate cells, achieved by promoting VAMP2 degradation, impairing SNARE complex formation, blocking FPN translocation, and subsequently causing iron overload and lipid peroxidation (44). Ellagic acid thus exerts hepatoprotective effects against liver diseases (especially MASLD) through antioxidant, anti-inflammatory, and anti-fibrotic activities, highlighting its potential as a medicinal and edible resource (45).

Polysaccharides from *Astragalus mongholicus, Lycium barbarum, Polygonatum sibiricum, Pueraria lobata*, and *Codonopsis pilosula* have drawn research interest for their potential to prevent and treat hepatic fibrosis. *Pueraria lobata* polysaccharides exert multi-targeted effects against hepatic fibrosis by alleviating oxidative stress and suppressing inflammatory damage in mouse liver tissue, as well as modulating the gut microbiota (46). *Polygonatum sibiricum* polysaccharide alleviates hepatic fibrosis by inhibiting the TGF-β/Smad signaling pathway through its anti-inflammatory and antioxidant effects (47). *Lycium barbarum* polysaccharides mitigate hepatic fibrosis, curb inflammation, and down-regulate the TLR/NF-κB axis (48). *Codonopsis pilosula* polysaccharides can effectively alleviate oxidative stress and inflammatory responses by regulating TLR4/NF-κB and TGF-β1/Smad3 signaling pathways, thereby attenuating hepatic fibrosis (49). Astragalus polysaccharide ameliorates hepatic fibrosis induced by alcohol through suppression of the TLR4/JNK/NF-κB/MyD88 pathway to alleviate inflammatory responses (50). Other bioactive compounds of *Astragalus mongholicus*, such as astragalus flavonoids and cycloastragenol, have also been studied. *Astragalus flavonoids* alleviate hepatic fibrosis by targeting the IKKβ/NF-κB signaling pathway, thereby suppressing inflammation (51). Cycloastragenol exerts anti-inflammatory and antioxidant actions via the TGF-β1/Akt signaling pathway, thereby alleviating hepatic fibrosis (52).

Meanwhile, it was found that some bioactive compounds from medicinal-food homologous plants exhibit therapeutic efficacy against hepatic fibrosis. Geniposide from *Gardenia jasminoides* alleviates hepatic fibrosis by inhibiting oxidative stress, inflammatory response, and apoptosis, as well as modulating metabolic pathways including glycerophospholipid, arginine, and proline metabolism (53). Geniposide alleviates high-fat diet-induced hepatic fibrosis in mice by activating the INSR-IRS2-Akt insulin signaling pathway and inhibiting inflammation (54). Trillin from *Dioscorea opposita* L. alleviates inflammation and thereby reduces hepatic fibrosis by modulating the NF-κB and TGF-β/Smad signaling pathways (55). Corosolic acid, isolated from Crataegus pinnatifida, exerts an anti-hepatofibrotic effect by alleviating inflammation through modulation of the TGF-β1/Smad2, NF-κB, and AMPK signaling axis (56). Bergapten from *Angelica dahurica* alleviates hepatic fibrosis by activating the FXR signaling pathway and thereby attenuating the inflammatory response (57). Imperatorin, another major active component of *Angelica dahurica*, alleviates hepatic fibrosis by inhibiting inflammation, angiogenesis, and the TGF-β signaling pathway (58). Dehydrotrametenolic acid methyl ester (ZQS5029-1) from *Wolfiporia cocos* alleviates NASH-related hepatic fibrosis by targeting Caspase-1 to suppress NLRP3 inflammasome activation, thereby inhibiting inflammation and HSC activation (59). Loganin, the main active component of *Cornus officinalis*, alleviates MCD diet-induced hepatic fibrosis in mice by inhibiting NLRP3 inflammasome activation (60). Hyperoside alleviates hepatic fibrosis by regulating the PARP-1-HMGB1 signaling pathway (61). Mogrosides, the main active components of *Siraitia grosvenorii*, alleviate NASH-associated hepatic fibrosis by inhibiting oxidative stress and inflammatory progression (62). Naringin, the main active component of *Citrus aurantium* var. amara, alleviates hepatic fibrosis through metabolic/inflammatory pathways (63) and by disrupting the VEGF-C mediated hepatocyte-macrophage axis to reduce pro-fibrotic macrophage infiltration (64). Taraxasterol, an active component of *Taraxacum mongolicum*, alleviates hepatic fibrosis by exerting anti-inflammatory effects and regulating multiple signaling pathways including Hippo, HIF-1α, TGF-β/Smad, and Wnt (65).

In addition to the above studies on defined active compounds, a considerable body of evidence for medicinal-food homologous plants against hepatic fibrosis comes from crude extracts. These crude extract studies typically prepare samples by water or ethanol extraction, characterize the main chemical constituents using techniques such as liquid chromatography-mass spectrometry, and then validate the overall pharmacodynamic effects. For example, *Lonicera japonica* water extract, rich in chlorogenic acid and other polyphenols, effectively prevents hepatic fibrosis through its triple action of antioxidant, anti-inflammatory, and anti-apoptotic effects (66, 67). Chlorogenic acid reduces oxidative stress/inflammation via PI3K/AKT/mTOR (68) and inhibits HSC activation/collagen deposition via miR-21/TGF-β1/Smad7 (69). *Platycodon grandiflorus* aqueous extract attenuates hepatic fibrosis by inducing Nrf2-mediated antioxidant enzymes and suppressing oxidative stress and inflammation (70, 71). At the same time, the known active monomer platycodin D from *Platycodon grandiflorus* has been reported to alleviate hepatic fibrosis by inhibiting NF-κB and iNOS expression and attenuating oxidative stress (72). *Portulaca oleracea* L. extract (POL-1) attenuates hepatic fibrosis via mediation of TLR4/NF-κB, TGF-β1/Smad2, and Bcl-2/Bax pathways (73). An 8-week randomized controlled trial showed that *Portulaca oleracea* supplementation significantly improved hepatic fibrosis in NAFLD patients, reducing liver stiffness and hepatorenal ultrasound index (74). Fructus Mori aqueous extracts (MFAEs) exert a pronounced protective effect against hepatic fibrosis by activating the Nrf2/HO-1 signaling pathway to suppress hepatic oxidative stress and inflammation (75). Mulberry leaf extract (MLE; Folium Mori) ameliorates hepatic fibrosis by activating the Nrf2-dependent antioxidant defense pathway (76). 1-Deoxynojirimycin, the main active component of mulberry leaf, alleviates diabetes-related hepatic fibrosis by activating the AMPK/SIRT1 pathway and reducing oxidative stress (77). *Chrysanthemum morifolium* aqueous extract alleviates steatosis-associated hepatic fibrosis by activating pan-PPAR pathways and suppressing inflammatory responses (78). Although these crude extract-based studies have not further isolated the material to single active components, they are supported by chemical analysis data (e.g., mass spectrometry) and thus provide reliable pharmacodynamic evidence for the anti-fibrotic application of medicinal-food homologous plants.

Numerous studies have indicated that bioactive compounds derived from medicinal-food homologous plants can alleviate hepatic fibrosis by enhancing antioxidant capacity and suppressing inflammatory responses, primarily by targeting key pathways, encompassing TGF-β/Smad and TLR/NF-κB. Despite these promising findings, the current body of research exhibits notable limitations. The majority of studies rely on chemically-induced animal models, particularly models of hepatic fibrosis, which poorly recapitulate the complex etiology and pathophysiology of human chronic liver diseases such as alcohol-induced liver disease or MASH. Furthermore, most studies employ only male animals, and few investigate dose-response relationships or long-term safety. More notably, research focus remains highly concentrated on a limited number of compounds (e.g., hesperidin, puerarin). A recent study revealed that hesperetin contributes to the amelioration of hepatic fibrosis by improving the composition of the gut microbiota and regulating autophagy in HSC (79). Evidently, with the continuous advancement in research on hesperetin's anti-hepatic fibrosis phenotypes, the research on hesperetin has become increasingly systematic. Based on this substantial body of pre-clinical evidence, initiating relevant clinical studies has become a critical next step that warrants further efforts. It is critical to note that the pathological state of hepatic fibrosis significantly alters the pharmacokinetic profile of puerarin. This must be considered a key factor when evaluating its therapeutic efficacy. Moreover, this principle extends to the experimental design of all studies investigating drug candidates for hepatic fibrosis and the subsequent interpretation of their efficacy data.

## 4 Inhibition of hepatic stellate cell activation and apoptosis induction

**Figure 3:**
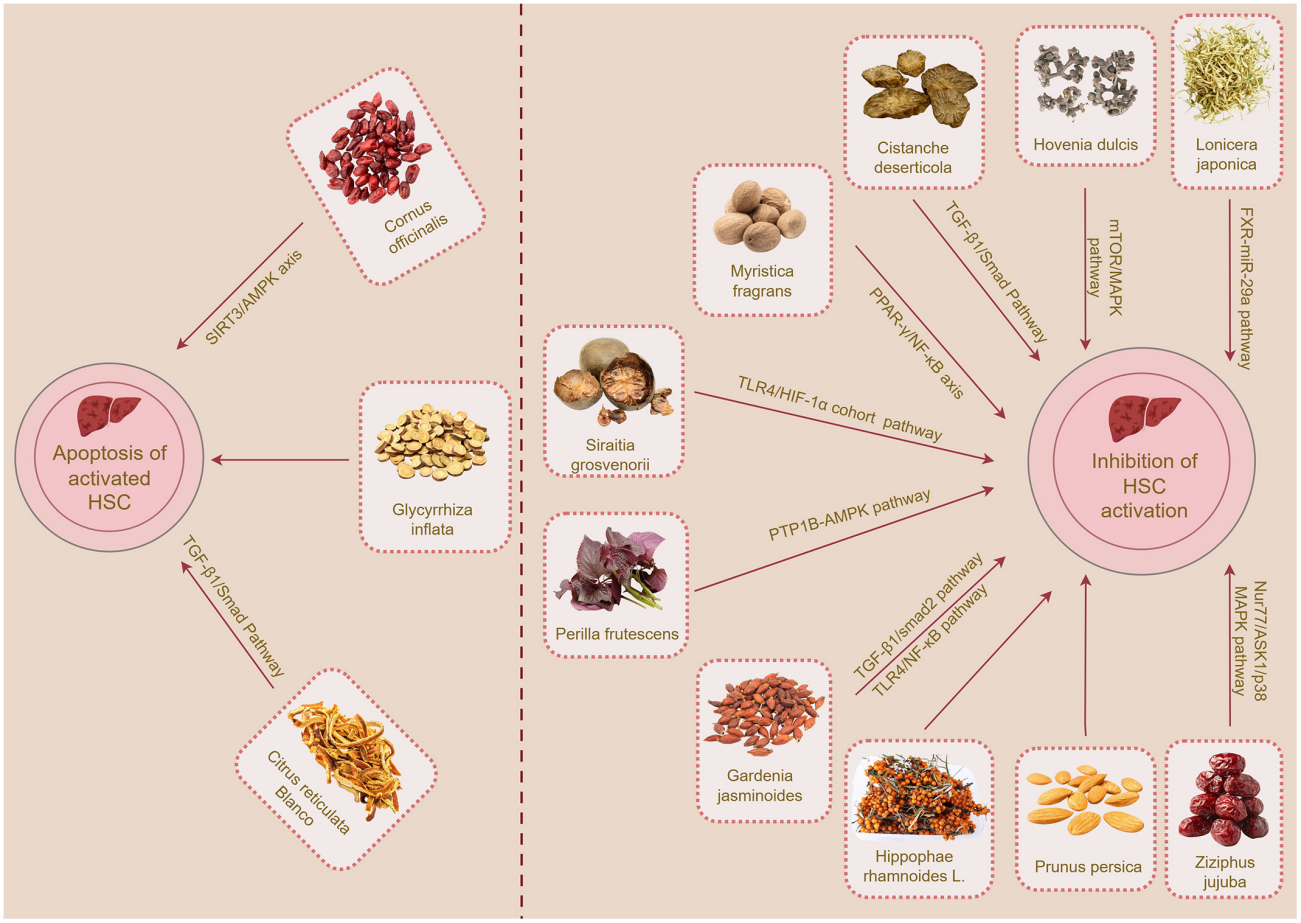
Medicinal-food homologous (MFH) plants ameliorate hepatic fibrosis by targeting hepatic stellate cell activation and apoptosis.

The activation of quiescent HSC represents the cornerstone of hepatic fibrosis. While inhibiting the initiation of this activation process is a fundamental therapeutic approach (as discussed in the previous section), it is equally critical to address the population of already-activated myofibroblasts. These cells are responsible for the excessive deposition of ECM that characterizes fibrosis. The induction of apoptosis specifically in activated HSC serves as this essential clearance mechanism, working in concert with activation inhibitors to reverse established fibrosis and restore liver architecture. This section summarizes the bioactive components, derived from medicinal-food homologous plants, that function by suppressing HSC activation or promoting apoptosis (Figure 3).

Several components inhibit HSC activation. Spinosin from *Ziziphus jujuba* alleviates hepatic fibrosis by curbing activated HSC via the Nur77/ASK1/p38 MAPK pathway (80). Puerarin has been extensively studied in the field of hepatic fibrosis. Its anti-activation effect on HSC is mediated by specific inhibition of the TGF-β1/Smad pathway, thereby exerting an anti-hepatofibrotic effect (81). Identification of bioactive compounds in *Hippophae rhamnoides* revealed 46 components that suppress HSC activation to mitigate hepatic fibrosis (82). Some of the research data on *Gardenia jasminoides* illustrate a compelling trajectory from documenting the efficacy of a crude extract to identifying its specific active constituents. An initial study showed that the crude extract alleviates hepatic fibrosis by targeting the TGF-β1/Smad2 pathway in HSC (83). Subsequently, a novel pectin-like polysaccharide (GJE-0.2-2) was found to inhibit HSC activation via the TLR4/NF-κB pathway (84). Additionally, geniposide, a main component of *G. jasminoides*, suppresses HSC activation through the Sonic hedgehog (Shh) signaling pathway (85). Mogroside IVE, the main active component of *Siraitia grosvenorii*, alleviates hepatic fibrosis in mice by inhibiting the TLR4/HIF-1α signaling pathway (86). Dihydromyricetin alleviates hepatic fibrosis by inducing autophagy in HSC and enhancing NK cell-mediated killing, thereby inhibiting HSC activation (87). Total flavonoids from *Hovenia dulcis* seeds alleviate hepatic fibrosis by inhibiting the PI3K/AKT signaling pathway, suppressing HSC proliferation and migration, promoting apoptosis, and reducing α-SMA and collagen I expression (88). In parallel, methoxyeugenol from *Myristica fragrans* deactivates HSC to attenuate hepatic fibrosis through the PPAR-γ/NF-κB axis (89). Luteolin-7-diglucuronide, a novel PTP1B inhibitor from *Perilla frutescens*, suppresses HSC activation to reduce hepatic fibrosis via PTP1B-AMPK pathways (90). Furthermore, sweroside from Lonicera japonica presents a unique mechanism of action by engaging the nuclear receptor FXR and its downstream miRNA regulator, miR-29a (91). Chlorogenic acid alleviates NASH-associated hepatic fibrosis by promoting PGC1α/NRF1-mediated mitochondrial biogenesis and by suppressing HSC activation and HMGB1-induced ECM production via the TGFβ-Smad2/3 pathway (92). Platycodin D alleviates hepatic fibrosis by activating the JNK/c-Jun signaling pathway to inhibit HSC activation (93). Mulberry marc anthocyanins alleviate hepatic fibrosis in rats by inhibiting HSC activation and reducing α-SMA expression (94). Botanical extracts such as Semen Persicae (the seed of *Prunus persica*) extract also exhibit efficacy, attenuating HSC activation and hepatic fibrosis (95). Amygdalin, the main active component of peach seed, alleviates hepatic fibrosis by inhibiting mTOR/S6K1-mediated PDCD4 degradation, upregulating PDCD4 expression, and thereby suppressing the JNK/c-Jun pathway and HSC activation (96). It also blocks the TGF-β1/Smad2/3 and NF-κB p65 signaling pathways (97). Fucoidan from *Laminaria japonica* alleviates alcoholic hepatic fibrosis by inhibiting the TLR4/NF-κB pathway and HSC activation (98), and its oligosaccharides block the JAK/STAT3/FUT8 axis to suppress TGF-β/Smad signaling (99).

Other components pre-dominantly induce apoptosis of activated HSC. Hesperetin (100) and 18β-glycyrrhetinic acid (101) also induce HSC apoptosis to exert anti-hepatic fibrosis effects. Glycyrrhetinic acid, the main active component of licorice, alleviates hepatic fibrosis by upregulating miR-663a and inhibiting the TGF-β/Smad signaling pathway, thereby suppressing HSC activation (102). It is worth noting that glycyrrhizic acid, glycyrrhetinic acid, and their clinical formulation GLPS (18α-/18β-glycyrrhetinic acid) are effective against hepatic fibrosis and other liver diseases (103). Hyperoside exerts anti-fibrotic effects by inducing apoptosis of HSC, inhibiting the NF-κB signaling pathway, and reducing α-SMA and collagen levels (104).

A number of MFH-derived compounds exert dual functions, suppressing HSC activation while simultaneously promoting their apoptosis. Phenylethanol glycosides from *Cistanche deserticola* and dihydromyricetin from *Hovenia dulcis* suppress HSC activation and induce HSC apoptosis via the TGF-β1/Smad and mTOR/MAPK pathways, respectively (105, 106). High-pressure wine-steaming (HPWS) of *Cornus officinali*s produces the strongest anti-fibrotic effect, suppressing HSC activation and inducing HSC apoptosis via the SIRT3-AMPK axis (107). Morroniside from *Cornus officinalis* targets GATA3 and LAL to inhibit HSC activation (108); its pro-apoptotic role warrants further study. *Ganoderma lucidum* polysaccharide extracted from sporoderm-removed spores alleviates hepatic fibrosis by inhibiting HSC activation and inducing apoptosis of activated HSCs via the TLR4/NF-κB/MyD88 and TGF-β/Smad pathways (109). The hepatoprotective effects of *Ganoderma lucidum* polysaccharide have been extensively studied across various liver diseases, demonstrating additional mechanisms such as anti-inflammatory, antioxidant, and gut microbiota-modulating activities (110). *Dendrobium officinale* polysaccharide alleviates hepatic fibrosis by modulating the gut-liver axis, inhibiting the LPS-TLR4-NF-κB pathway, and inducing apoptosis of activated HSCs (111); it also suppresses the SMO/Gli1 pathway and angiogenesis (112). In a TAA-induced hepatocellular carcinoma model, echinacoside not only ameliorated hepatic fibrosis but also improved the survival rate of HCC model rats (113), suggesting potential dual actions that merit further investigation.

This section summarizes the bioactive compounds from medicinal-food homologous plants that exert anti-hepatofibrotic effects by inhibiting HSC activation or inducing their apoptosis. However, current research pre-dominantly focuses on inhibiting activation, whereas investigations into alleviating hepatic fibrosis by inducing HSC apoptosis remain incomplete. In fact, an ideal anti-hepatofibrotic strategy should possess a dual function: preventing the activation of quiescent HSC and simultaneously eliminating activated HSC. Inducing apoptosis is a key approach to achieving the latter. Given the close phenotypic linkage and potential synergy between inhibition of activation and promotion of apoptosis, systematically investigating the HSC apoptosis pathway is crucial. Such research will not only fill the current knowledge gap but also be of paramount importance for developing comprehensive and effective anti-hepatic fibrosis therapies. However, despite this promising early finding, research on *Hippophae rhamnoides* L has since stagnated, with no subsequent large-scale trials (114). It is worth noting that traditional processing techniques, such as high-pressure wine-steaming for *Cornus officinalis*, can significantly enhance efficacy, providing a practical basis for optimizing the application of medicinal-food homologous plants. It is noted that systematic toxicological and pharmacokinetic studies are lacking for most bioactive components, and effective doses often lack sufficient pre-clinical safety support. The practice of referring to safety dose ranges from authorities like EFSA has yet to become widespread.

## 5 Modulation of the gut microbiota

**Figure 4:**
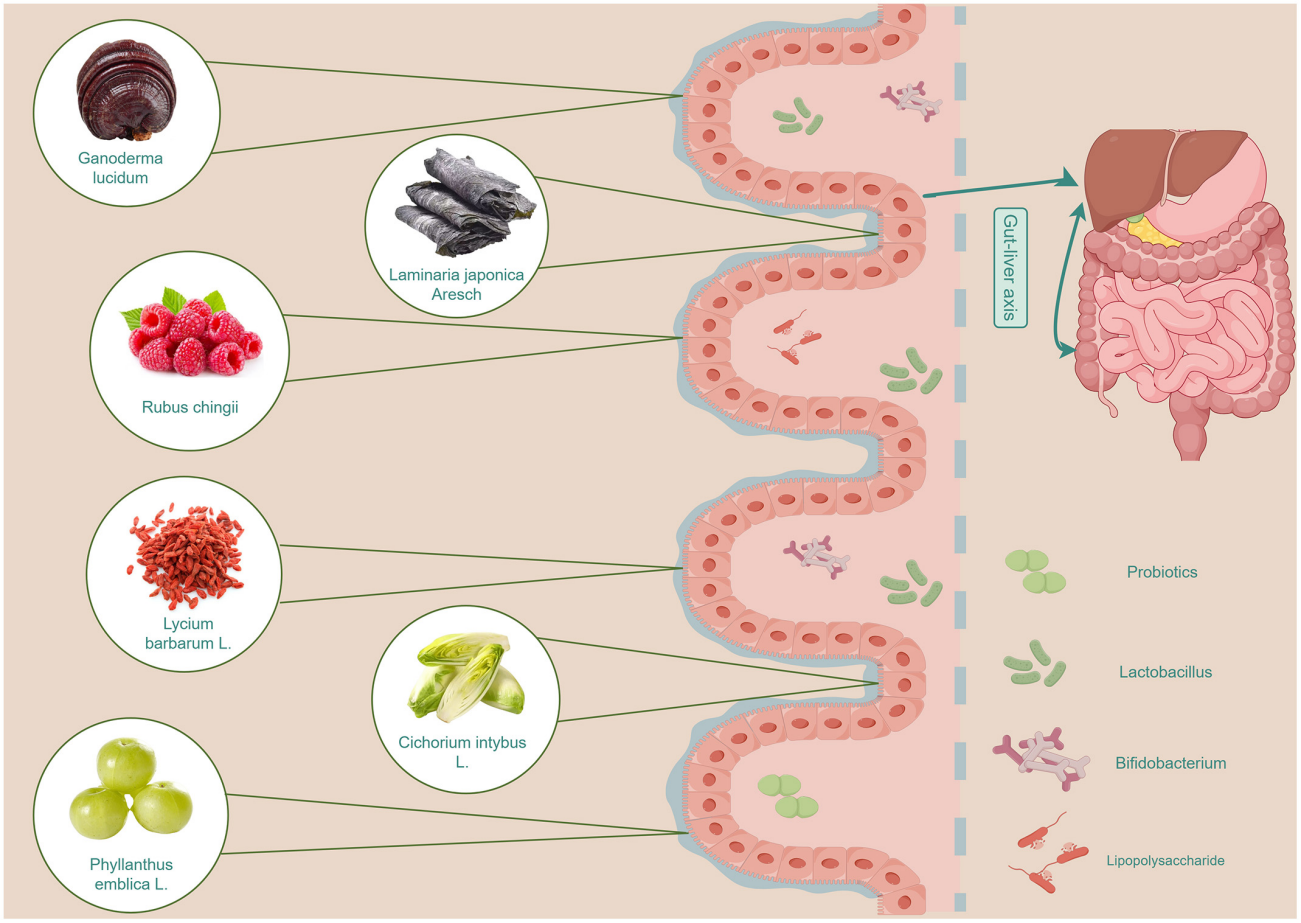
Medicinal-food homologous (MFH) plants ameliorate hepatic fibrosis via modulating the gut-liver axis.

A critical advantage of many MFH plants is their primary action within the intestinal milieu. There is a bidirectional regulatory relationship between medicinal-food homologous plants and gut microbiota; they not only regulate the homeostasis of the microbiota but also undergo biotransformation by the microbiota, thereby influencing the ultimate physiological effects (115). This section will explore the mechanisms by which MFH plants improve gut microbiota dysbiosis to alleviate fibrosis, primarily through the modulation of the gut-liver axis (Figure 4).

Specifically, multiple bioactive compounds have been shown to exert anti-liver-fibrosis effects by regulating the gut microbiota and the gut-liver axis. For instance, hyperoside attenuates NASH-associated hepatic fibrosis by modulating Flot2/TLR4 signaling and pyroptosis, and by restoring the gut-liver axis through gut microbiota modulation and LPS reduction (116). Ursolic acid alleviates hepatic fibrosis by inhibiting the NOX2/NLRP3 pathway and modulating gut microbiota (117). Dihydromyricetin alleviates hepatic fibrosis in mice by reshaping the gut microbiota and host metabolism, thereby improving the inflammatory response (118). A low-molecular-weight oligosaccharide (LBO) from *Lycium barbarum* L. exerts anti-hepatofibrotic effects by reshaping gut microbiota and improving mitochondrial function (119). A novel fructan, LRMP1, isolated from *Lycium ruthenicum*, alleviates hepatic fibrosis in mice by modulating gut microbiota. It inhibits hepatocyte ferroptosis via microbiota-dependent post-biotic signaling, unveiling a natural polysaccharide–gut–liver axis with therapeutic promise (120). PLP2, a homogeneous water-soluble polysaccharide from *Pueraria montana* var. lobata, suppresses ferroptosis via the gut microbiota-dependent Nrf2/HO-1/GPX4 axis, supporting its clinical use (121). A separate fruit peptidoglycan acts by dampening the TGF-β/Smad7 pathway, a mechanism likewise associated with microbiota remodeling (122). Moreover, lactucin from *Cichorium intybus* L. has been reported to mitigate hepatic fibrosis via modulating the TLR4-MyD88-MAPK/NF-κB axis through gut microbiota (123), and via regulating gut microbial metabolites (acetic acid and butyric acid) to improve enterohepatic circulation and modulating Stat3/TGF-β1 signaling pathways through the gut-liver axis (124).

Beyond specific bioactive compounds, several crude extracts also demonstrate efficacy through gut microbiota-mediated mechanisms. *Ganoderma lucidum* extracts (total triterpenoids) can alleviate hepatic fibrosis by enhancing the interaction between metabolites and g_Ruminococcus through modulation of the NF-κB and TGF-β1/Smad signaling pathways (125). The extract of Cistanche pentaphylloside from *Cistanche deserticola* alleviates hepatic fibrosis by regulating the gut-liver axis (126). *Rubus chingii* unripe fruits extract plays a role in alleviating hepatic fibrosis by improving the associated gut microbiota imbalance through the TGF-β/Smads signaling pathway (127). Additionally, Aqueous extract of *Phyllanthus emblica* L. (AEPE) retards hepatic fibrosis in NAFLD mice by reshaping the gut microbiota (128). They function as prebiotics to reshape gut microbiota and directly enhance intestinal barrier integrity, thereby reducing the translocation of enteric toxins to the liver (129). Fucoidan from *Scytosiphon lomentaria* ameliorates alcohol-induced liver fibrosis by increasing the abundance of *Parabacteroides distasonis* and modulating the NF-κB/MAPK and Nrf2 signaling pathways (130). This represents a natural interventive mechanism that is challenging for many chemical drugs to replicate.

Notably, low molecular weight *Lycium barbarum* oligosaccharide (LBN) exemplifies how structural refinement, overcoming the limitations of conventional high molecular weight polysaccharides can enhance prebiotic activity and bioactivity. This highlights the importance of not only identifying active components but also optimizing their physicochemical properties for greater biological efficacy.

## 6 Therapeutic potential of spice extracts in hepatic fibrosis

**Figure 5:**
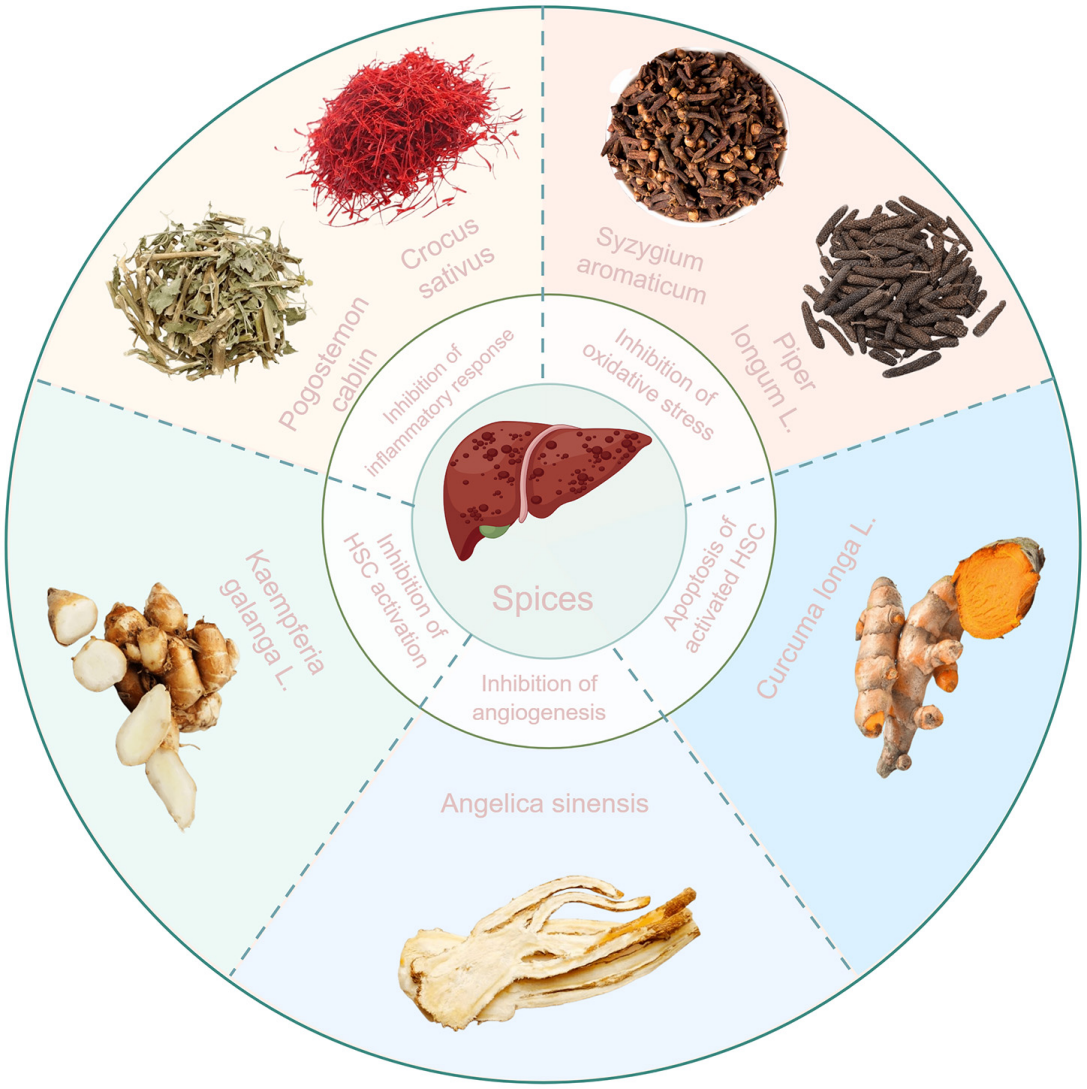
Spice extracts ameliorate hepatic fibrosis through a multi-targeted approach.

Beyond their culinary role in enhancing flavor, spices have been integral to traditional medicine systems worldwide for millennia, epitomizing the concept of Medicinal-Food Homology. These MFH plants often target pathological mechanisms of fibrosis, such as oxidative stress, inflammation, and HSC activation (Figure 5). Piperlongumine, an extract of *Piper longum* L., inhibits inflammation, oxidative-nitrosative stress and HSCs activation by modulating TGF-β1/Smad pathway and EMT pathways, thereby alleviating hepatic fibrosis (131). Pogostone from *Pogostemon cablin* alleviates hepatic fibrosis due to non-alcoholic fatty liver disease by inhibiting NLRP3 inflammasomes (132). *Crocus sativus* L. and its constituent Crocin ameliorate hepatic fibrosis through anti-inflammatory mechanisms mediated by Akt/HIF-1α/VEGF signaling pathways and PPAR-γ, respectively (133, 134). Kaempferol from *Kaempferia galanga* L. alleviates hepatic fibrosis by promoting ASIC1a-eIF2α-ATF-4 signaling pathway to reduce hepatic stellate cell activation (135). Levistilide A from *Angelica sinensis* exhibits a multi-faceted approach against hepatic fibrosis. In addition to its established role in hindering angiogenesis via the VEGF signaling pathway (136), recent research demonstrates that it also directly attenuates hepatic stellate cell activation by inhibiting the NF-κB/iNOS/NO signaling pathway (137). *Angelica sinensis* polysaccharide suppresses HSC activation by the IL-22/STAT3 axis (138). As early as 2020, a systematic review summarized the pre-clinical research on Curcuma (an extract of *Curcuma longa* L.) in hepatobiliary diseases (139). Subsequent meta-analyses have further confirmed the definitive therapeutic effect of curcumin on liver cirrhosis (140, 141). Currently, there are already many curcumin products available for everyday anti-inflammation and liver protection. The therapeutic effects of curcumin on hepatic fibrosis have recently been summarized (142). Beyond curcumin, sesquiterpenoids in Curcuma also exhibit significant anti-hepatofibrotic activity. Recent research indicates that the enhanced anti-hepatofibrotic effect of vinegar-processed *Curcumae Rhizoma* is mediated through the PI3K/Akt/mTOR signaling pathway, with key bioactive sesquiterpenes including furanodiene, curcumol, and curdione (143). Furthermore, curcumol can directly inhibit hepatic fibrosis by Sirt1/NICD pathway-mediated endoplasmic reticulum stress triggers necroptosis in HSC (144).

These findings preliminarily confirm the potential of spice-derived compounds to exert anti-hepatofibrotic effects by targeting core pathways such as inflammation and oxidative stress, highlighting their value as a resource for medicinal and dietary applications. However, this field faces significant limitations; the current methodological approaches are indistinguishable from those used in general botanical drug research, failing to account for the unique attributes of spices as dietary components. This uniqueness is reflected in the fundamental disparity between daily dietary intake (characterized by low doses and long-term exposure) and experimental models (which employ high doses of purified compounds). A paradigm shift in future research is urgently needed. Studies should prioritize long-term intervention experiments using dietary-relevant doses and investigate the pharmacological activity of whole extracts and compounds, thereby accurately defining the unique role of spices in anti-hepatofibrotic strategies. *Carthamus tinctorius* L. (safflower) is a traditional medicinal herb that also finds uses in culinary practices, such as a natural coloring agent, rather than as a spice. For example, a study showed that the mechanism by which its active compound, Hydroxysafflor yellow A, modulates the miR-29a-3p/PDGFRB axis, underscores its therapeutic potential rather than its dietary role (145). Therefore, research on MFH plants must adhere to a fundamental principle: the investigation should be grounded in their inherent dual attributes for both dietary and medicinal use.

## 7 Combined application

In addition to single active compounds and crude extracts, combination therapies involving natural products have shown synergistic effects against hepatic fibrosis, often outperforming monotherapy. For example, luteolin, a flavonoid found in honeysuckle, chrysanthemum, and perilla, combined with silibinin attenuated thioacetamide-induced liver fibrosis in a rat model (146). In MASH models, co-administration of tetrahydrocurcumin (THU) and the ALK-5 inhibitor EW-7197 attenuated hepatic fibrosis more effectively than either monotherapy (147). *Syzygium aromaticum* extract combined with Silymarin alleviated hepatic fibrosis, with the combination showing superior efficacy to monotherapy, via TLR4/MyD88/NF-κB pathway (148). Oral administration of *Ziziphus jujuba* seed powder combined with seed oil alleviated hepatic fibrosis more effectively than either agent alone, via synergistic inhibition of the TGF-β/Smad and NF-κB pathways (149). Taken together, these findings suggest that rationally designed combinations of natural products or their co-administration with established agents can produce synergistic anti-fibrotic effects, offering a promising strategy for enhancing therapeutic efficacy while potentially reducing adverse effects.

## 8 Critical reappraisal of current evidence

### 8.1 Cross-mechanism correlation analysis

The three anti-fibrotic mechanisms, anti-oxidation/anti-inflammation, HSC targeting, and gut-liver axis modulation, are interconnected. Oxidative stress and inflammation drive HSC activation; MFH compounds (e.g., hesperetin, puerarin, polysaccharides) break this cycle (34, 35, 39, 40, 46–50). Direct HSC-targeting agents (curcumin, spinosin, 18β-glycyrrhetinic acid) inhibit activation or induce apoptosis, stopping the inflammation-fibrosis feedback (80, 101, 140–142). Gut dysbiosis promotes LPS/TLR4 signaling, worsening both inflammation and HSC activation; certain MFH plants (*Lycium barbarum* oligosaccharides, Ganoderma triterpenoids, lactucin) reshape gut microbiota to counter these processes (119, 123, 125). Many MFH compounds act via multiple mechanisms, e.g., puerarin (39, 40, 81) and *Lycium barbarum* polysaccharides (48, 119, 122). Thus, early or mild fibrosis may benefit from antioxidant or gut-modulating MFH plants, whereas advanced fibrosis likely requires direct HSC-targeting agents, possibly in combination or sequential regimens. However, head-to-head comparisons among different mechanistic classes are lacking, and the late YAP/TAZ mechanosignaling stage remains unexplored, which are key gaps for future research.

### 8.2 Analysis of dose variability factors

As shown in Table 2, effective doses of MFH compounds vary considerably across studies. Key factors include molecular properties and administration route. Flavonoid aglycones absorb better due to lipophilicity, whereas glycosides require deglycosylation by intestinal enzymes (150, 151). Many flavonoids and terpenoids are efflux transporter substrates, reducing oral bioavailability (152). Polysaccharides have very low intestinal absorption due to their large molecular size and thus act mainly indirectly via the gut-liver axis (153, 154); their effective doses vary greatly (e.g., 50–100 mg/kg for Codonopsis polysaccharide vs. 400–1,600 mg/kg for *Lycium barbarum* polysaccharide) (155). Some glycosylated terpenoids (e.g., certain saponins) may require higher doses due to poor absorption, though exceptions exist (e.g., Platycodin D with potent low-dose efficacy) (156). Route of administration matters: puerarin is effective at 100–200 mg/kg intraperitoneally but up to 800 mg/kg orally, and hepatic fibrosis further reduces its oral bioavailability via P-gp/UGT upregulation (41); however, most compounds have only oral data available, making broad comparisons difficult. Model differences (CCl4, TAA, BDL, HFD, MCD) may affect pharmacokinetics of active compounds, as seen with hesperidin in TAA/BDL/CCl4 models (35–38) and puerarin in CCl4/DMN models (39, 40, 81). In summary, molecular weight, lipophilicity, efflux transport, gut microbiota dependence, model-induced pharmacokinetic changes, and administration route are core factors explaining dose variability.

**Table 2:** Summary of dose-effect relationships of major MFH bioactive compounds in pre-clinical studies of hepatic fibrosis.

Compound	Model (*in vivo*/*in vitro*)	Effective dose range (*in vivo*)	Effective dose range (*in vitro*)	References
1. Flavonoids and phenolics				
1.1. Flavonoid aglycones				
Kaempferol	CCl4-induced rats; HSC-T6	12.5–50 mg/kg;	5, 10, 20 μm	(91)
Hesperetin	HFD-induced rats; HepG2 cells	100–300 mg/kg;	2.5–10 μm	(1)
Dihydromyricetin	CCl4-induced mice; LX2 cells	100 mg/kg	10–50 μm	(51, 52, 86)
1.2. Flavonoid glycosides				
Spinosin	CCl4-induced mice; AML12/LX2 cells	20–40 mg/kg	10–20 μm	(46)
Naringin	MCD/HFD-induced mice	25–200 mg/kg	/	(38, 39)
Luteolin-7-diglucuronide	CCl4-induced mice; HSCs/LX2 cells	50–100 mg/kg	5–50 μm	(57)
Hesperidin	CCl4/TAA/BDL/DMN-induced rats	100–200 mg/kg	/	(2–5)
Hyperoside	CCl4-induced mice; LX2 cells	100–200 mg/kg	0.5–2 μm	(17, 68)
Puerarin	CCl4/DMN-induced rats/mice	200–800 mg/kg	/	(6, 7, 58, 102)
1.3 Phenolic acids and other phenolics				
Chlorogenic acid	CCl4-induced rats; LX2 cells	15–60 mg/kg	20–80 μg/ml	(23, 24)
Echinacoside	TAA-induced rats	15–60 mg/kg	/	(45)
Ellagic acid	Iron/TiO2-induced mice; AML12/L02 cells	25–100 mg/kg	≤20–45 μm	(33, 34)
Tetrahydrocurcumin	MCD-induced mice; LX2 cells	100 mg/kg	1 μm	(99)
2. Polysaccharides and peptidoglycans				
*Codonopsis pilosula* Polysaccharide	CCl4-induced mice; LX2 cells	50–100 mg/kg	50–200 μg/ml	(9)
*Gardenia jasminoides* polysaccharide	CCl4-induced mice; LX2 cells	50–100 mg/kg	0.1–1.0 mg/ml	(54)
*Lycium barbarum* peptidoglycan	CCl4-induced mice; LX2 cells	50–200 mg/kg	0.25–1.0 mg/ml	(78)
Fucoidan	CCl4/alcohol-induced mice	100–300 mg/kg	/	(72, 73, 87)
*Ganoderma lucidum* polysaccharide	CCl4-induced mice; HSC-T6	150–300 mg/kg	1.25–5 mg/ml	(69)
Astragalus polysaccharide	Alcohol-induced rats	200–400 mg/kg	/	(12)
*Pueraria lobata* polysaccharide	CCl4-induced mice	200–400 mg/kg	/	(79)
*Angelica sinensis* polysaccharide	CCl4-induced mice	200 mg/kg	/	(92)
*Lycium barbarum* oligosaccharides	CCl4-induced mice	200 mg/kg	/	(76)
*Dendrobium officinale* polysaccharide	CCl4-induced rats; HSC-T6	200–800 mg/kg	100–400 μg/ml	(70, 71)
*Lycium barbarum* polysaccharides	CCl4-induced rats	400–1,600 mg/kg	/	(10)
*Polygonatum sibiricum* polysaccharide	HFD + ethanol + CCl4-induced rats	800–1,600 mg/kg	/	(11)
3. Terpenoids				
3.1 Non-glycosylated terpenoids				
Taraxasterol	CCl4-induced mice	2.5–10 mg/kg	/	(41)
Corosolic acid	HC/CCl4-induced mice; HepG2/LX2 cells	10–20 mg/kg	5–20 μm	(16)
*Cichorium intybus* lactucin	CCl4-induced rats; HSC-T6/RAW264.7 cells	2.5–10.5 mg/kg	5–25 μg/ml	(80, 81)
18 beta-glycyrrhetinic acid	BDL/CCl4-induced mice; LX2 cells	20–50 mg/kg	20–80 μm	(65, 66)
Ursolic acid	MCD/CCl4-induced mice	40 mg/kg	/	(85)
Dehydrotrametenolic acid methyl ester	HFD + CCl4-induced mice	30–75 mg/kg	5–20 μm	(20)
Curcumol	CCl4-induced mice; LX2 cells	30 mg/kg	20–100 μm	(97)
Cycloastragenol	CCl4-induced mice	50–200 mg/kg	/	(14)
3.2 Glycosylated terpenoids				
Platycodin D	BDL/CCl4-induced mice; LX2 cells	1–8 mg/kg	20–80 μm	(28, 49)
Loganin	MCD-induced mice	5–30 mg/kg	/	(37)
Mogroside IVE	CCl4-induced mice; HSC-T6	25 mg/kg	0.5–10 μm	(42)
Crocin	CCl4-induced rats	20–80 mg/kg	/	(89, 90)
Geniposide	CCl4/HFD-induced mice; HSC-T6	50–150 mg/kg	25–100 μm	(35, 56)
Sweroside	CCl4-induced mice; LX2/L02 cells	125 mg/kg	50 μm	(47)
4. Coumarins				
Imperatorin	CCl4-induced rats; LX2 cells	15–25 mg/kg	100 μm	(19)
Bergapten	CCl4-induced mice; HSC-T6/LX2 cells	100–300 mg/kg	3.125–12.5 μm	(18)
5. Other small molecules				
Methoxyeugenol	CCl4-induced mice; HepG2 cells	0.25–1.0 mg/kg	15–250 μm	(43)
Piperlongumine	BDL-induced mice	1.25–2.5 mg/kg	/	(88)
Amygdalin	CCl4-induced rats; LX2 cells	3–100 mg/kg	1.25–100 μm	(61, 62)
Levistilide A	CCl4-induced rats/mice; LX2/RAW264.7 cells	3–9 mg/kg	12.5–50 μm	(93, 94)
Pogostone	HFD-induced mice; primary hepatocytes cells	5–20 mg/kg	50–200 μg/ml	(95)
Trillin	CCl4-induced mice	50–100 mg/kg	/	(15)
6. Herbal extracts and mixtures				
*Platycodon grandiflorum* water extract	BDL/DMN-induced rats	10–100 mg/kg	/	(26, 27)
*Gardenia jasminoides* extract	BDL-induced rats; LX2 cells	25–100 mg/kg	20–80 μmol/L	(55)
*Portulaca oleracea* extract	CCl4-induced mice; LX2 cells	50–200 mg/kg	30–100 μg/ml	(29)
Fructus Mori aqueous extract	CCl4-induced mice; HepG2 cells	100–200 mg/kg	20 μg/ml	(25)
*Chrysanthemum morifolium* water extract	MCD-induced mice; HepG2 cells	200–400 mg/kg	1–10 μg/ml	(32)
*Hovenia dulcis* total flavonoids	CCl4-induced mice; HSC-T6	150–450 mg/kg	5–30 μg/ml	(53)
Mulberry marc anthocyanins	CCl4-induced rats	200–800 mg/kg	/	(50)
*Rubus chingii* unripe fruit extract	CCl4-induced mice	450–900 mg/kg	/	(83)
*Lonicera japonica* water extract	CCl4/TAA-induced mice/rats	200–2,500 mg/kg	5–100 μg/ml	(21, 22)
*Phyllanthus emblica* extract	CDAHFD-induced mice	900–3,600 mg crude drug/kg	/	(75)

### 8.3 From experimental doses to dietary reality: a translational gap

A key question is whether pre-clinical effective doses of MFH plants can be achieved through normal dietary intake. As shown in Table 3, our calculated human equivalent doses (HED) for most compounds are far above typical daily intake. Therefore, therapeutic effects are not achievable by diet alone without enhanced delivery or concentrated extracts. Even for compounds like honeysuckle-derived chlorogenic acid, effective levels are only marginally reachable with very high daily consumption (e.g., strong herbal decoction). Thus, standardized extracts, nutraceuticals, or functional foods are required for therapy. Nevertheless, long-term low-dose dietary intake may offer cumulative preventive benefits, supporting the development of products like supplements with enhanced bioavailability. Future studies should prioritize long-term low-dose dietary interventions with clear reporting of active compound content in foods.

**Table 3:** Translational feasibility: human equivalent dose vs. dietary intake.

Compound	Pre-clinical oral dose (mg/kg)	HED (60 kg adult, mg/day)	Dietary source and typical intake	Feasibility (diet alone)	Recommended role	References
Hesperetin	100–300 mg/kg (rat)	972–2,916	Citrus fruits; typical dietary intake low	Not achievable	Prevention (diet); treatment (extract)	(1)
Hesperidin	100–200 mg/kg (rat)	972–1,944	Orange juice: 235–407 mg/L → ~70–120 mg per 240 ml serving	Not achievable	Prevention (diet); therapy (extract)	(2–5)
Puerarin	100- 800 mg/kg (rat)	972–7,776	Kudzu root (dietary use as food ingredient or starch); content ~32 mg/g → estimated daily intake typically < 50 mg	Not achievable (requires excessive dried root intake)	Standardized extract (nutraceutical)	(6, 7, 58, 102)
Chlorogenic acid	15–60 mg/kg (rat)	145.8–583.2	Honeysuckle: ~27 mg/g dry flower; 5–15 g herb/day as tea → 136–408 mg/day	Marginally achievable (only with high daily intake as herbal tea)	Dietary prevention	(23, 24)
Naringin	25–200 mg/kg (mouse)	121.5–972	Grapefruit juice: 307–376 mg/L → ~50–100 mg/glass	Not achievable	Prevention (diet); therapy (extract)	(38, 39)
Fucoidan	100–300 mg/kg (mouse)	486–1,458	Brown seaweeds (e.g., haidai): ~1%−2.5% dry weight → < 10 mg/day	Not achievable	Dietary supplement; therapeutic extract	(72, 73, 87)
*Lycium barbarum* polysaccharides	400–1,600 mg/kg (rat)	3,888–15,552	Goji berry: polysaccharides ~5%−10% dry weight → 500–2,000 mg/day (10–20 g berries)	Not achievable	Dietary supplement; therapeutic extract	(10)
Glycyrrhetinic acid	20–50 mg/kg (mouse)	97.2–243	Licorice root (as glycyrrhizin, hydrolyzed in gut); intake variable	Feasible but risky (hypertension risk)	Therapeutic (caution)	(65, 66)

Human equivalent dose (HED) was calculated according to FDA (2005) guidance using the body surface area method, with conversion factors of 0.162 for rats and 0.081 for mice.

All HED values refer to total daily dose for a 60 kg adult.

Typical dietary intakes are estimates based on literature values and common serving sizes; actual intake can vary significantly depending on food source, processing methods, and population dietary habits.

## 9 Toward clinically-oriented research strategies and standards

### 9.1 Stage-specific intervention of MFH plants across hepatic fibrosis progression

Hepatic fibrosis evolves from an early inflammatory stage, during which Kupffer cells are activated and HSCs initiate without significant ECM deposition (157). It then progresses to a mid-stage of HSC perpetuation with active proliferation and collagen production, and ultimately to a late cirrhotic stage characterized by matrix stiffening and the YAP/TAZ mechanotransduction loop (158, 159). Current MFH research pre-dominantly targets early-to-mid events. In the early stage, compounds such as hesperetin, puerarin, and polysaccharides from Astragalus or Lycium suppress oxidative stress and inflammation via Nrf2 and TLR4/NF-κB pathways, thereby preventing HSC initiation (34, 35, 39, 48–50). In the mid-stage, spinosin, morroniside, 18β-glycyrrhetinic acid, and curcumol inhibit activated HSC proliferation or induce their apoptosis through Nur77/ASK1/p38 MAPK, SIRT3-AMPK, PRDX1/2-ROS, and Sirt1/NICD pathways (80, 101, 107, 108, 144). However, for the late cirrhotic stage dominated by YAP/TAZ mechanosignaling and positive stiffness feedback, no MFH compound has been rigorously validated, representing a critical knowledge gap (30, 32, 33). Future studies should adopt stage-specific animal models and long-term low-dose regimens to clarify whether MFH plants can prevent, reverse, or only delay fibrosis progression at different phases.

### 9.2 A disease-to-drug screening strategy

Previous reviews have primarily categorized medicinal-food homologous compounds by chemical structure (160, 161), limited their scope to specific compound classes such as flavonoids (162), or focused on particular components (e.g., iridoids) from a single plant like *Gardenia jasminoides* across a broad spectrum of liver diseases (163). While systematic, these approaches face a common challenge: they are inefficient for identifying the most therapeutically advantageous compounds for a specific condition—such as hepatic fibrosis—given the diverse pathological states of liver diseases (fibrosis, steatosis, hepatocellular carcinoma). In contrast, the present review adopts a disease-to-drug approach, explicitly targeting hepatic fibrosis. We systematically evaluate existing evidence and screen the most promising candidate compounds based on evidence strength, targeting capability, therapeutic efficacy, and clinical translation potential. This strategy is designed to enhance drug discovery efficiency and increase the success rate of clinical translation.

### 9.3 Ensuring translational value: research standards

This study's uniqueness lies in its rigorous methodology. We strictly adhered to the national “Catalog of Substances Traditionally Used as Both Food and Chinese Materia Medica,” including only legally edible varieties and parts (e.g., Eucommia leaves, Ganoderma fruiting bodies). Some studies fail to specify edible parts (e.g., seeds vs. peduncles), causing confusion. We also focused on natural compounds (e.g., hesperetin) rather than synthetic derivatives when basic pharmacology is unclear (164–168). This ensures safety as food while investigating health benefits, establishing their value in preventive healthcare, which is distinct from pure drugs or ordinary foods. While this may limit literature scope, it guarantees relevance to real dietary scenarios, enhancing translational value. We identify under-investigated yet promising resources (e.g., Dendrobium species; denbinobin (169)) and highlight a critical contradiction: the disconnect between long-term low-dose dietary intake in populations and high-dose interventions with purified compounds in pre-clinical models.

## 10 Limitations and future directions

The insights from this review must be considered within the context of its limitations. The following section outlines these constraints and proposes corresponding strategic priorities for future research to enhance the field's translational impact.

### 10.1 Uneven mechanistic coverage

Current research in the treatment of hepatic fibrosis with MFH plants exhibits a notable imbalance in phenotypic coverage, with the majority of studies focusing on oxidative stress, inflammation response, the activation of hepatic stellate cells, and gut microbiota modulation, while mechanisms such as autophagy, apoptosis, ferroptosis, and pyroptosis remain relatively understudied. To advance a more comprehensive understanding of underlying mechanisms, future investigations should strengthen exploration of these underrepresented areas.

### 10.2 Scarcity of clinical evidence

Current evidence on MFH substances for hepatic fibrosis is largely pre-clinical; clinical data remain scarce. A few small studies exist (74, 114, 140, 141). This limits accurate assessment of real-world efficacy and safety. Rigorously designed, adequately powered randomized controlled trials (RCTs) using standardized MFH extracts and validated endpoints (e.g., liver stiffness, FIB-4, histology) are urgently needed. Future trials should prioritize dose-escalation studies for safety and tolerability, pharmacokinetic profiling in fibrotic patients to account for disease-altered drug disposition (as shown with puerarin), and combination studies to leverage synergistic effects of multi-compound extracts.

### 10.3 Realistic dosing: prevention vs. treatment

The current literature remains unclear whether MFH plants prevent fibrosis onset or reverse established fibrosis. Future research should develop realistic dosing regimens reflecting real-world intake, including long-term, low-dose intervention studies with dietary-relevant levels. A stage-specific strategy should be adopted: low-dose dietary MFH intake for primary prevention in healthy or at-risk individuals, and high-dose standardized extracts for therapeutic intervention in diagnosed fibrosis, with careful safety monitoring. Rigorous dose-finding and pharmacokinetic studies in fibrotic patients (as exemplified by puerarin (41)) are critical to bridge the pre-clinical-clinical gap.

### 10.4 High-quality research for overcoming critical barriers

The field lacks high-quality evidence, as about one-third of studies still use crude extracts with undefined bioactive compounds. This impedes mechanistic analysis, reproducibility, and clinical translation. Future research should prioritize identifying key bioactive compounds via systematic fractionation using modern chromatographic and spectroscopic techniques. Comparative studies between refined compounds and crude extracts can clarify contributions of specific molecules. Subsequently, their direct molecular targets and signaling pathways must be elucidated to unravel the multi-target mechanisms.

### 10.5 From evidence to product development

The evidence base of this review is limited by small studies and a narrow focus on legal MFH parts, possibly excluding other valuable research. To translate findings into practice, future research should prioritize product development. Following curcumin's success, MFH active components have great potential for functional foods and personalized regimens. Their advantages, including long safe use, low toxicity, nanotechnology enhanced bioavailability, and omics guided personalization, make this approach promising. Incorporating these compounds into foods or beverages can provide sustainable, compliant strategies for long term liver disease management and anti fibrotic intervention.

### 10.6 Safety assessment gaps

**Figure 6:**
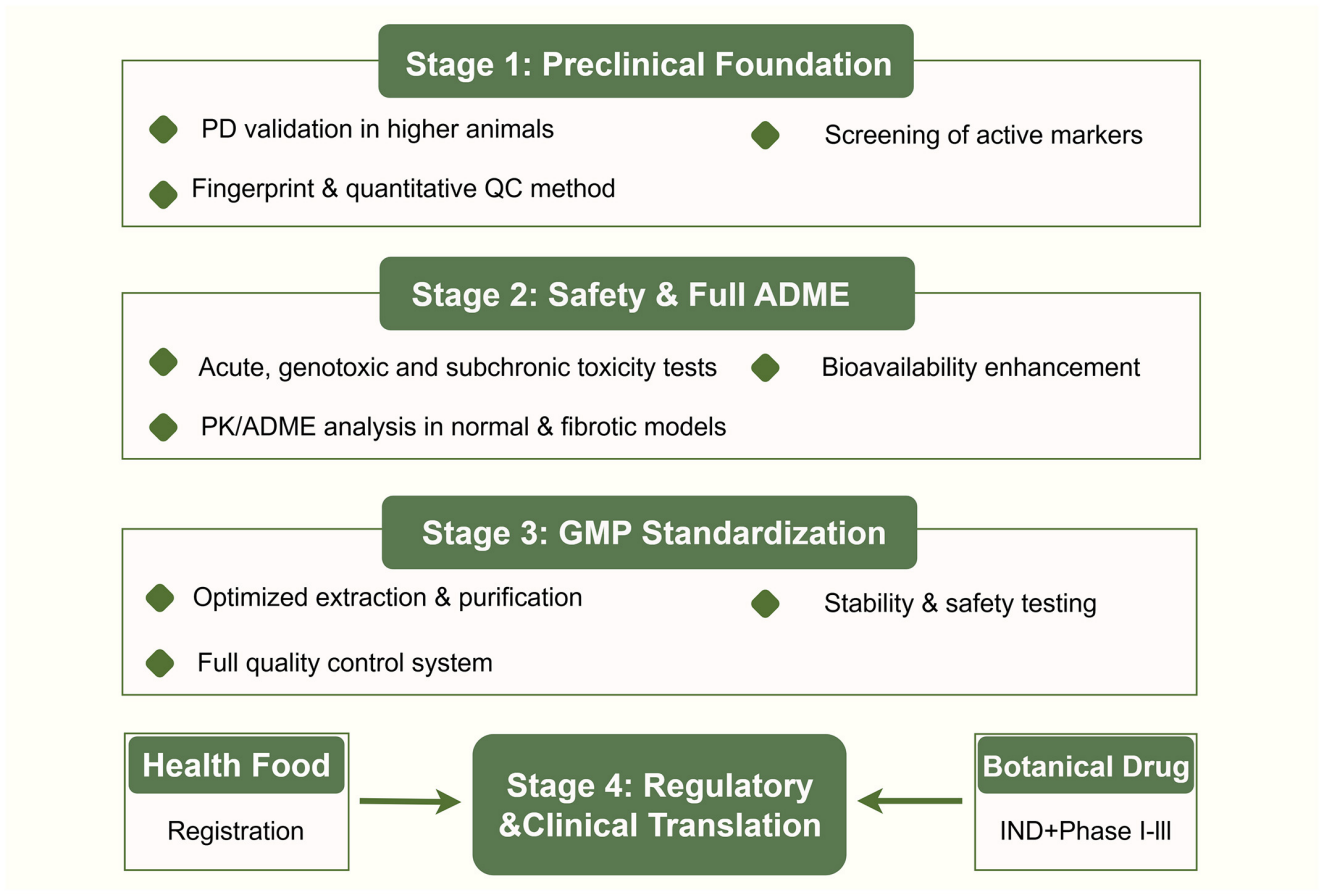
Multi-stage pathway for safety evaluation and regulatory translation of MFH plants in hepatic fibrosis. Stages 1–3 provide core pre-clinical, ADME/toxicity, and GMP data. Depending on the intended product positioning, the generated data can support either a health food registration **(left)** or a botanical drug IND and clinical trials **(right)**. Fast approval refers to regulatory pathways with reduced timelines for well-characterized food-derived substances. PD, pharmacodynamics; QC, quality control; ADME, absorption, distribution, metabolism, excretion; PK, pharmacokinetics; GMP, good manufacturing practices; IND, investigational new drug; Phase I-III, clinical phase I-III.

Despite the traditional presumption of safety for MFH plants, systematic safety evaluation in hepatic fibrosis is lacking. Most pre-clinical studies report effective doses without toxicological data (e.g., NOAEL, LOAEL). Hepatic fibrosis can alter drug metabolism, potentially increasing toxicity risk (41). Beyond safety, the absence of PK and metabolic stability data is a major translational hurdle. Most MFH compounds lack key PK parameters (bioavailability, half-life, tissue distribution) and metabolic stability data (phase II conjugation, gut microbiota metabolism). Chronic use of undefined extracts raises concerns about hepatotoxicity, nephrotoxicity, and herb-drug interactions (170–172). Long-term toxicology and clinical safety trials are lacking. Moreover, authoritative safety data (e.g., JECFA, EFSA, FDA GRAS) are missing for most compounds in Table 1, preventing reliable HED calculation and dietary feasibility assessment. Thus, future research must prioritize comprehensive PK profiling of lead MFH compounds in fibrotic animal models to enable rational dosing and reliable HED calculation. To bridge this gap, we constructed a multi-stage pathway (Figure 6) covering pre-clinical validation, ADME/toxicity, GMP standardization, and regulatory translation, drawing on ICH, EFSA, and FDA guidance. This roadmap supports generating necessary safety data.

### 10.7 Underexplored combination regimens

Evidence on combination regimens involving MFH plants is very limited. Although a few studies reported synergistic effects (146–149), systematic investigations of MFH plants combined with conventional anti-fibrotic drugs or with each other are lacking. Given the multi-pathogenic nature of hepatic fibrosis, rational combinations targeting distinct mechanisms may improve efficacy and reduce toxicity. Future research should prioritize well-designed combination studies, including MFH plants with direct HSC-targeting agents, gut-microbiota modulators, other MFH plants, approved anti-fibrotic agents (e.g., obeticholic acid, resmetirom), or conventional hepatoprotective drugs (e.g., ursodeoxycholic acid), along with dose optimization and safety evaluation.

## 11 Conclusion and perspectives

In summary, the value of studying medicinal-food homologous plants for treating hepatic fibrosis is found not in supplanting potent end-stage therapies, but in addressing the significant unmet need for prevention and early-stage intervention throughout the prolonged course of chronic liver disease. This review consolidates evidence that various bioactive compounds and extracts derived from MFH plants hold great potential for alleviating hepatic fibrosis through multi-target mechanisms and phenotypes. We systematically elaborate on their anti-hepatofibrotic effects through three mechanisms: anti-oxidative stress and anti-inflammation response, suppressing HSC activation and inducing their apoptosis, and rebalancing gut microbiota to restore gut-liver axis function. By adopting a more critical, clinically relevant, and nutritionally grounded research framework, as advocated in this review, we can truly unlock their potential and establish their unique role in the prevention and management of chronic liver diseases and hepatic fibrosis. Consequently, this research direction not only addresses an unmet clinical need for safe, long-term strategies but also leverages the distinctive strengths of the MFH plants, offering a promising pathway to bridge the gap between dietary prevention and pharmacological treatment in hepatic fibrosis care.
